# Synthesis, pharmacology and molecular docking on multifunctional tacrine-ferulic acid hybrids as cholinesterase inhibitors against Alzheimer’s disease

**DOI:** 10.1080/14756366.2018.1430691

**Published:** 2018-02-06

**Authors:** Jie Zhu, Hongyu Yang, Yao Chen, Hongzhi Lin, Qi Li, Jun Mo, Yaoyao Bian, Yuqiong Pei, Haopeng Sun

**Affiliations:** a Department of Medicinal Chemistry, China Pharmaceutical University, Nanjing, China;; b School of Pharmacy, Nanjing University of Chinese Medicine, Nanjing, China;; c School of Nursing, Nanjing University of Chinese Medicine, Nanjing, China

**Keywords:** Alzheimer’s disease, cholinesterase inhibitor, multi-target ligand, tacrine-ferulic hybrid, molecular docking

## Abstract

The cholinergic hypothesis has long been a “polar star” in drug discovery for Alzheimer’s disease (AD), resulting in many small molecules and biological drug candidates. Most of the drugs marketed for AD are cholinergic. Herein, we report our efforts in the discovery of cholinesterases inhibitors (ChEIs) as multi-target-directed ligands. A series of tacrine-ferulic acid hybrids have been designed and synthesised. All these compounds showed potent acetyl-(AChE) and butyryl cholinesterase(BuChE) inhibition. Among them, the optimal compound **10g**, was the most potent inhibitor against AChE (electrophorus electricus (eeAChE) half maximal inhibitory concentration (IC_50_) = 37.02 nM), it was also a strong inhibitor against BuChE (equine serum (eqBuChE) IC_50_ = 101.40 nM). Besides, it inhibited amyloid β-protein self-aggregation by 65.49% at 25 μM. In subsequent *in vivo* scopolamine-induced AD models, compound **10g** obviously ameliorated the cognition impairment and showed preliminary safety in hepatotoxicity evaluation. These data suggest compound **10g** as a promising multifunctional agent in the drug discovery process against AD.

## Graphical abstract

**Figure:**
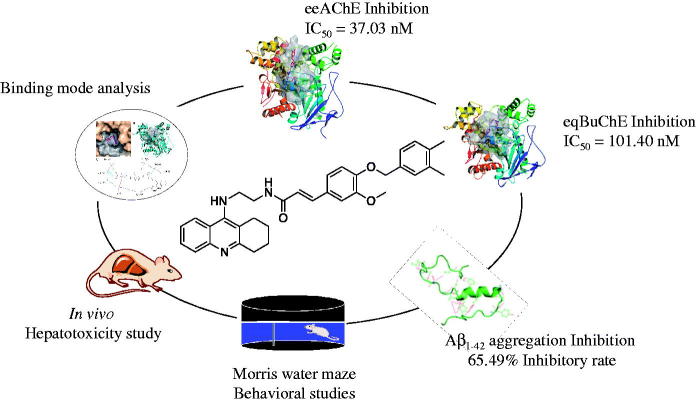

## Introduction

Alzheimer’s disease (AD) is one of the major threats to the whole world. Every 3 s one new case is diagnosed and the number will almost double every 20 years^1^. AD is estimated to cost the world 818 billion dollars in 2015 alone and by 2030 the cost will rise to two trillion^2^. These numbers are struggling. Currently, the number of drugs against AD is five in all^3^. Commercial drugs for AD treatment are unable to prevent or halt Alzheimer’s disease, so it is necessary to develop novel compounds with potential therapeutic value in this day and age^4–7^. Multi-target-directed ligands (MTDLs), aiming to simultaneously regulate multiple pathological processes in the development of neurodegenerative cascade, were developed as a promising therapeutic approach^8–11^.

Cholinergic system, which plays an important role in the regulation of learning, cognition, and memory processes^12^, has been extensively studied for the design of anti-Alzheimer’s drugs^13^ ^,^ ^14^. Currently, three of the drugs approved for AD treatment target on cholinesterase, including donepezil, rivastigmine, and galantamine. The most promising approach for the symptomatic treatment of AD is to increase the synaptic levels of acetylcholinesterase (AChE) in the brain^15^ ^,^ ^16^. At the neuronal level, AChE can be hydrolysed by two types of cholinesterases (ChEs): acetylcholinesterases (AChEs) and butyrylcholinesterases (BuChEs)^17^. During the early stage of AD, AChE plays a dominant role in AChE hydrolysis while BuChE plays only a supportive role^18^ ^,^ ^19^. So, inhibiting the activity of AChE is an effective way to prevent AD progression. In advanced AD, the activity of AChE decreases to 10–15% of normal values in certain brain regions, while the amount of BuChE remains the same or even increases up to two-fold^20^. Hence, inhibiting the activity of AChE encounters ineffectiveness during later stages of AD. Evidence showed that BuChEs can rescue the cholinesterase function in the absence of AChE. Therefore, a balance between AChE and BuChE inhibition will be more beneficial^21–23^.

Previously, our group has disclosed a series of tacrine-cinnamic hybrids^24^. The structure-activity relationship (SAR) analysis on cinnamic acid revealed that the introduction of 3-OMe-4-OBn to the benzene ring of cinnamic acid (4-Bn ferulic acid, compound **10a**) was the best. So, structural modification on the cinnamic acid moiety, which is replaced by ferulic acid with different substitutions deserved further research. In the present study, we designed a series of tacrine-ferulic acid hybrids with structural modification on benzyl moiety against both AChEs and BuChEs for the treatment of AD, in which MTDLs approach was applied. The target compounds were synthesised and evaluated for their ChEs inhibitory activities with optimal compounds tested for their inhibitory effects on self-induced Aβ1–42 aggregation. The SAR of synthesized compounds was discussed. Furthermore, two optimal compounds **10d** and **10g** were tested in AD mice model for the *in vivo* behavioral and hepatotoxic evaluations.

## Methods

### Chemistry

#### General experimental

All chemicals, reagents, and solvents were purchased from commercial companies. When necessary, solvents were used with further purification and dryness. Reactions were monitored by analytical thin layer chromatography (TLC) on silica gel 60 F254 precoated plates (purchased from Qingdao Haiyang Inc., China). Spots were visualized by ultraviolet light at 254 and 365 nm. Column chromatography was performed on silica gel (200–300 mesh) for the purification of intermediates and final compounds. Melting points were determined using a Mel-TEMP II melting point apparatus. ^1^H NMR and ^13 ^C NMR spectra were recorded on a Bruker Avance (300 MHz for ^1^H; 500 MHz for ^13 ^C, Billerica, MA) at 300 K dissolved in deuterated dimethyl sulfoxide(DMSO-d_6_) or deuterated chloroform (CDCl_3_) with tetramethylsilane (TMS) as an internal standard. NMR data were analysed by MestReNova software (Mestrelab Research, S.L., Spain). Chemical shifts were reported in ppm (*δ*), Coupling constants (*J*) were given in hertz, and peak multiplicities were reported as singlet (s), doublet (d), triplet (t), and multiplet (m). High-resolution mass spectrometry (HRMS) was performed on a Mariner Mass Spectrum (ESI) or an LC/MSD TOF HR-MS Spectrum. Final compounds were named following IUPAC rules as applied by ChemDraw Professional (version 15.0, Darmstadt, Germany).

#### General procedures for the synthesis and spectral data of the synthesised compounds

*General procedure for the synthesis of 2-aminobenzoic acid (* ***2*** *).* To a solution of Sodium hydroxide of 2N normality (2 N × NaOH; 40 ml), compound **1** (10 g) was added and stirred at room temperature overnight. The mixture was acidified with concentrated hydrochloric acid (HCl) until pH = 4–5. The precipitate was collected by filtration, washed with cold water, and dried over an infrared lamp, resulting in compound **2** as a white solid and used in the next step without further purification. The total yield of compound **2** obtained was 8.9 g (97.4%).

*General procedure for the synthesis of 9-chloro-1,2,3,4-tetrahydroacridine (* ***3*** *).* To a mixture of 2-aminobenzoic acid (compound **2**; 5 g, 36.2 mmol) and cyclohexanone (3.8 ml, 36.2 mmol) in a three-necked round bottom flask equipped with mechanical stirrer, additionally a funnel and thermometer was placed and 15 ml of Phosphoryl chloride (POCl_3_) was added placing the flask on ice bath. The mixture was allowed to reflux for 3 h and then was poured onto ice. The resulting mixture was filtered through a Celite pad and the filtrate was extracted with Dichloromethane (CH_2_Cl_2_; 3 × 15 ml) and the organic layers were washed with brine, dried over anhydrous sodium sulphate (Na_2_SO_4_). After evaporation *in vacuo*, the resulting residue was purified on silica gel chromatograph (PE/EA = 8:1) to furnish a pale brown solid compound **3**. The total yield of compound **3** obtained was 5.99 g (76.0%).

*General procedure for the synthesis of N^1^–(1,2,3,4-tetrahydroacridin-9-yl)ethane-1,2-diamine (* ***4*** *).* Ethylenediamine (3 ml, 45.94 mmol) and sodium iodide (catalytic amount) were added to 10 ml of 1-pentanol and heated to 160 °C. Then, a solution of Compound **3** (2 g, 9.19 mmol) in 30 ml 1-pentanol was added dropwise via an additional funnel to the above mixture at 160 °C. After being stirred at 160 °C for 18 h, the resulting mixture was quenched by the addition of water, later the solution was acidified to pH = 4 with concentrated HCl. The mixture was stirred at room temperature for 30 min. The aqueous phase was separated and basified with solid NaOH until pH = 13–14 and extracted with CH_2_Cl_2_ (3 × 15 ml). The CH_2_Cl_2_ layer was then washed with brine and dried over anhydrous Na_2_SO_4_. After concentration, the crude product was purified by silica gel column chromatograph (CH_2_Cl_2_/methanol(MeOH)/triethylamine(Et_3_N) = 60:1:0.3) to give compound **4** as a brown oil. The total yield of compound **4** obtained was 0.6750 g (30.4%).

*General procedure for the synthesis of benzyl (E)-3–(4-(benzyloxy)-3-methoxyphenyl)acrylate (* ***7a*** *–* ***7m*** *).* Compound **5** (0.5 g, 2.57 mmol) and potassium carbonate (K_2_CO_3_; 1.42 g, 10.30 mmol) were added to 15 ml of DMF and stirred at room temperature for 15 min. Compound **6** was added dropwise to the above mixture solution. After being stirred at 82 °C for 4 h, the reaction mixture was quenched with water. The precipitate was filtrated and the filter cake was washed with water to give the crude product which could be used in the next step without further purification.

*General procedure for the synthesis of (E)-3–(4-(benzyloxy)-3-methoxyphenyl)acrylic acid (* ***8a*** *–* ***8m*** *).* To a mixture solution of 2 N x NaOH (30 ml) and MeOH (30 ml) compound **7a**–**7 m** was added. The reaction mixture was heated to reflux for 3 h. Then, MeOH in the solution was removed and the pH was adjusted to around 2 by adding concentrated HCl. The precipitate was filtrated, washed with cold water, and dried over an infrared lamp to get compound **8a**–**8m**.

*General procedure for the synthesis of (E)-3–(4-(benzyloxy)-3-methoxyphenyl)acryloyl chloride (* ***9a*** *–* ***9m*** *).* Thionyl chloride (SOCl_2_; 3 ml, 27.57 mmol) was added to a solution of compound **8a–8m** (0.93 mmol) in 5 ml of anhydrous CH_2_Cl_2_. After being refluxed for 3 h, the reaction mixture was evaporated to remove excess SOCl_2_. The residue was diluted with anhydrous CH_2_Cl_2_ for next step.

*General procedure for the synthesis of (E)-3–(4-(benzyloxy)-3-methoxyphenyl)-N-(2-((1,2,3,4-tetrahydroacridin-9-yl)amino)ethyl)acrylamide (* ***10a*** *–* ***10m*** *).* To a mixture solution of compound **4** (0.2 g, 0.84 mmol) and K_2_CO_3_ (0.26 g, 1.86 mmol) in anhydrous CH_2_Cl_2_ cooled to 0 °C compound **9a**–**9m** was added dropwise. The reaction mixture was stirred at room temperature overnight and quenched by the addition of water. The organic layer was separated, washed with brine, and dried over anhydrous Na_2_SO_4_. The solution was evaporated to afford the crude product. Then, the crude product was chromatographed on silica gel (CH_2_Cl_2_/MeOH/Et_3_N = 120:1:0.6) to get compound **10a**–**10 m**.

*(E)-3–(4-(benzyloxy)-3-methoxyphenyl)-N-(2-((1,2,3,4-tetrahydroacridin-9-yl)amino)ethyl)acrylamide (* ***10a*** *).* Yellow powder, yield: 28%, melting point (m.p.) 158–160 °C. ^1^H NMR (300 MHz, CDCl_3_): *δ* 7.93 (d, *J* = 8.0 Hz, 1H), 7.84 (d, *J* = 8.0 Hz, 1H), 7.67 (s, 1H), 7.58 (d, *J* = 15.5 Hz, 1H), 7.48–7.23 (m, 7H), 6.96 (s, 2H), 6.78 (d, *J* = 8.0 Hz, 1H), 6.43 (d, *J* = 15.4 Hz, 1H), 5.11 (s, 2H), 3.78 (s, 3H), 3.64 (s, 4H), 2.95 (s, 2H), 2.58 (s, 2H), and 1.76 (s, 4H). ^13 ^C NMR (500 MHz, CDCl_3_): *δ* 167.63, 158.27, 150.76, 149.83, 149.62, 146.93, 141.40, 136.56, 128.64, 128.48, 128.12, 128.05, 127.96, 127.25, 123.79, 122.71, 121.81, 119.83, 118.17, 116.02, 113.44, 110.36, 70.85, 55.96, 49.82, 40.68, 33.69, 25.05, 22.99, and 22.63. HRMS (ESI) *m/z* calculated for C_32_H_33_N_3_O_3_ [M + H]^+^ 508.2595 was found to be 508.2592.

*(E)-3–(4-((4-fluorobenzyl)oxy)-3-methoxyphenyl)-N-(2-((1,2,3,4-tetrahydroacridin-9-yl)amino)ethyl)acrylamide (* ***10b*** *).* Yellow powder, yield: 46%, m.p. 75–77 °C. ^1^H NMR (300 MHz, CDCl_3_): *δ* 7.95 (d, *J* = 8.4 Hz, 1H), 7.86 (d, *J* = 8.4 Hz, 1H), 7.57 (d, *J* = 15.5 Hz, 1H), 7.52–7.44 (m, 1H), 7.43–7.33 (m, 2H), 7.28 (d, *J* = 9.0 Hz, 1H), 7.11–6.90 (m, 4H), 6.80 (d, *J* = 8.7 Hz, 2H), 6.33 (d, *J* = 15.6 Hz, 1H), 5.08 (s, 2H), 3.82 (s, 3H), 3.66 (s, 4H), 2.98 (s, 2H), 2.66 (s, 2H), and 1.82 (s, 4H). ^13 ^C NMR (500 MHz, CDCl_3_): *δ* 163.24, 158.61, 156.97, 151.36, 147.18, 145.00, 144.95, 136.67, 127.59, 127.57, 124.53, 124.49, 124.44, 123.42, 119.22, 118.41, 117.14, 113.59, 110.89, 110.75, 108.80, 105.63, 65.51, 51.27, 45.42, 35.58, 27.52, 19.92, 18.26, 17.94, and 17.30. HRMS (ESI) *m/z* calculated for C_32_H_32_FN_3_O_3_ [M + H]^+^ 526.25 was found to be 526.2519.

*(E)-3–(4-((4-chlorobenzyl)oxy)-3-methoxyphenyl)-N-(2-((1,2,3,4-tetrahydroacridin-9-yl)amino)ethyl)acrylamide (* ***10c*** *).* Yellow powder, yield: 28%, m.p. 70–72 °C. ^1^H NMR (300 MHz, CDCl_3_): δ 7.98 (d, J = 8.4 Hz, 1H), 7.90 (d, J = 8.3 Hz, 1H), 7.60–7.51 (m, 2H), 7.43–7.29 (m, 6H), 7.02 (d, J = 7.2 Hz, 2H), 6.84 (d, J = 8.4 Hz, 1H), 6.28 (d, J = 15.6 Hz, 1H), 5.15 (s, 2H), 3.91 (s, 3H), 3.71 (s, 4H), 3.04 (s, 2H), 2.73 (s, 2H), and 1.88 (s, 4H). ^13 ^C NMR (500 MHz, CDCl_3_): *δ* 167.64, 158.03, 150.91, 149.65, 149.50, 146.68, 141.28, 135.08, 133.82, 128.81, 128.62, 128.57, 128.24, 127.86, 123.81, 122.77, 121.71, 119.70, 118.38, 115.87, 113.52, 110.40, 70.12, 55.94, 49.87, 40.68, 33.53, 25.02, 22.96, and 22.57. HRMS (ESI) *m/z* calculated for C_32_H_32_ClN_3_O_3_ [M + H]^+^ 542.2205 was found to be 542.2206.

*(E)-3–(4-((4-bromobenzyl)oxy)-3-methoxyphenyl)-N-(2-((1,2,3,4-tetrahydroacridin-9-yl)amino)ethyl)acrylamide (* ***10d*** *).* Yellow powder, yield: 31%, m.p. 73–75 °C. ^1^H NMR (300 MHz, CDCl_3_): *δ* 7.97 (d, *J* = 8.4 Hz, 1H), 7.90 (d, *J* = 8.4 Hz, 1H), 7.63–7.47 (m, 4H), 7.36–7.29 (m, 4H), 7.01 (d, *J* = 6.5 Hz, 2H), 6.83 (d, *J* = 8.4 Hz, 1H), 6.29 (s, 1H), 5.12 (s, 2H), 3.89 (s, 3H), 3.69 (s, 4H), 3.04 (s, 2H), 2.73 (s, 2H), and 1.88 (s, 4H). ^13 ^C NMR (500 MHz, CDCl_3_): *δ* 167.50, 158.45, 150.66, 149.66, 149.49, 147.10, 141.35, 135.62, 131.77, 128.90, 128.44, 128.33, 128.23, 123.82, 122.65, 121.96, 121.71, 119.93, 118.30, 116.18, 113.53, 110.39, 70.15, 55.94, 49.78, 40.75, 33.81, 25.09, 23.02, and 22.68. HRMS (ESI) *m/z* calculated for C_32_H_32_BrN_3_O_3_ [M + H]^+^ 586.17 was found to be 586.1696.

*(E)-3–(3-methoxy-4-((2-methylbenzyl)oxy)phenyl)-N-(2-((1,2,3,4-tetrahydroacridin-9-yl)amino)ethyl)acrylamide (* ***10e*** *).* Yellow powder, yield: 13%, m.p. 93–95 °C. ^1^H NMR (300 MHz, CDCl_3_): *δ* 7.98 (d, *J* = 8.5 Hz, 1H), 7.91 (d, *J* = 8.4 Hz, 1H), 7.61–7.52 (m, 2H), 7.41 (d, *J* = 6.6 Hz, 1H), 7.35 (t, *J* = 7.5 Hz, 1H), 7.28 (s, 1H), 7.23 (s, 2H), 7.14 (d, *J* = 10.2 Hz, 1H), 7.10–7.01 (m, 2H), 6.90 (d, *J* = 8.2 Hz, 1H), 6.26 (d, *J* = 15.5 Hz, 1H), 5.16 (d, *J* = 9.0 Hz, 2H), 3.89 (s, 3H), 3.70 (s, 4H), 3.05 (s, 2H), 2.75 (s, 2H), 2.40 (s, 3H), and 1.90 (s, 4H). ^13 ^C NMR (500 MHz, CDCl_3_): *δ* 167.58, 158.35, 150.74, 150.04, 149.79, 141.50, 136.44, 134.37, 130.42, 128.49, 128.30, 128.26, 127.98, 126.09, 126.05, 123.84, 122.68, 121.89, 118.09, 116.12, 113.46, 110.38, 69.48, 55.97, 49.80, 40.72, 25.07, 23.02, 22.66, and 18.93. HRMS (ESI) *m/z* calculated for C_33_H_35_N_3_O_3_ [M + H]^+^ 522.2751 was found to be 522.2753.

*(E)-3–(3-methoxy-4-((3-methylbenzyl)oxy)phenyl)-N-(2-((1,2,3,4-tetrahydroacridin-9-yl)amino)ethyl)acrylamide (* ***10f*** *).* Yellow powder, yield: 33%, m.p. 98–100 °C. ^1^H NMR (300 MHz, CDCl_3_): *δ* 7.98 (d, *J* = 8.4 Hz, 1H), 7.90 (d, *J* = 8.3 Hz, 1H), 7.55 (t, *J* = 12.9 Hz, 2H), 7.40–7.23 (m, 5H), 7.14 (d, *J* = 6.6 Hz, 1H), 7.02 (d, *J* = 5.9 Hz, 2H), 6.88 (d, *J* = 8.5 Hz, 1H), 6.26 (d, *J* = 15.5 Hz, 1H), 5.16 (s, 2H), 3.91 (s, 3H), 3.70 (s, 4H), 3.04 (s, 2H), 2.74 (s, 2H), 2.37 (s, 3H), and 1.89 (s, 4H). ^13 ^C NMR (500 MHz, CDCl_3_): *δ* 167.58, 158.28, 150.75, 149.95, 149.62, 146.93, 141.48, 138.35, 136.48, 128.81, 128.54, 128.49, 128.19, 127.95, 127.87, 124.34, 123.83, 122.68, 121.88, 119.85, 118.05, 116.09, 113.40, 110.27, 70.95, 55.96, 49.81, 40.70, 33.70, 25.06, 23.01, 22.65, and 21.44. HRMS (ESI) *m/z* calculated for C_33_H_35_N_3_O_3_ [M + H]^+^ 522.2751 was found to be 522.2755.

*(E)-3–(4-((3,4-dimethylbenzyl)oxy)-3-methoxyphenyl)-N-(2-((1,2,3,4-tetrahydroacridin-9-yl)amino)ethyl)acrylamide (* ***10g*** *).* Yellow powder, yield: 45%, m.p. 78–80 °C. ^1^H NMR (300 MHz, CDCl_3_): *δ* 7.96 (d, *J* = 8.3 Hz, 1H), 7.88 (d, *J* = 8.3 Hz, 1H), 7.58 (d, *J* = 15.6 Hz, 1H), 7.50 (t, *J* = 7.4 Hz, 1H), 7.31 (d, *J* = 7.4 Hz, 1H), 7.20 (s, 1H), 7.18–7.09 (m, 2H), 6.99 (d, *J* = 5.2 Hz, 2H), 6.85 (d, *J* = 8.8 Hz, 1H), 6.67 (s, 1H), 6.30 (d, *J* = 15.5 Hz, 1H), 5.11 (s, 2H), 3.84 (s, 3H), 3.67 (s, 4H), 3.00 (s, 2H), 2.68 (s, 2H), 2.25 (d, *J* = 1.6 Hz, 6H), and 1.84 (s, 4H). ^13 ^C NMR (500 MHz, CDCl_3_): *δ* 168.27, 155.15, 152.48, 150.05, 149.64, 143.62, 141.43, 136.88, 136.47, 133.90, 129.82, 129.62, 129.30, 128.68, 127.80, 124.87, 124.00, 123.40, 122.02, 118.18, 118.07, 114.01, 113.35, 110.27, 70.86, 56.02, 53.46, 50.39, 40.17, 24.54, 22.58, 21.84, 19.81, and 19.54. HRMS (ESI) *m/z* calculated for C_34_H_37_N_3_O_3_ [M + H]^+^ 536.2908 was found to be 536.2907.

*(E)-3–(3-methoxy-4-((4-methylbenzyl)oxy)phenyl)-N-(2-((1,2,3,4-tetrahydroacridin-9-yl)amino)ethyl)acrylamide (* ***10h*** *).* Yellow powder, yield: 38%, m.p. 92–94 °C. ^1^H NMR (300 MHz, CDCl_3_): *δ* 7.95 (d, *J* = 8.4 Hz, 1H), 7.87 (d, *J* = 8.3 Hz, 1H), 7.56 (d, *J* = 15.6 Hz, 1H), 7.48 (t, *J* = 7.5 Hz, 1H), 7.30 (d, *J* = 7.7 Hz, 3H), 7.16 (d, *J* = 7.8 Hz, 2H), 7.02–6.94 (m, 2H), 6.82 (d, *J* = 8.8 Hz, 1H), 6.69 (s, 1H), 6.30 (d, *J* = 15.6 Hz, 1H), 5.12 (s, 2H), 3.83 (s, 3H), 3.67 (s, 4H), 2.99 (s, 2H), 2.66 (s, 2H), 2.33 (s, 3H), and 1.83 (s, 4H). ^13 ^C NMR (500 MHz, CDCl_3_): *δ* 168.11, 156.02, 152.00, 149.95, 149.66, 144.58, 141.47, 141.45, 137.81, 133.51, 129.31, 127.85, 127.35, 125.79, 123.94, 123.20, 121.97, 118.55, 118.17, 114.55, 113.42, 110.33, 70.78, 56.03, 50.21, 40.30, 32.31, 24.67, 22.68, 22.05, and 21.22. HRMS (ESI) *m/z* calculated for C_33_H_35_N_3_O_3_ [M + H]^+^ 522.2751 was found to be 522.2749.

*(E)-3–(4-((4-(tert-butyl)benzyl)oxy)-3-methoxyphenyl)-N-(2-((1,2,3,4-tetrahydroacridin-9-yl)amino)ethyl)acrylamide (* ***10i*** *).* Yellow powder, yield: 15%, m.p. 78–80 °C. ^1^H NMR (300 MHz, CDCl_3_): *δ* 7.95 (d, *J* = 8.3 Hz, 1H), 7.87 (d, *J* = 8.3 Hz, 1H), 7.56 (d, *J* = 15.6 Hz, 1H), 7.49 (d, *J* = 7.4 Hz, 1H), 7.41–7.28 (m, 5H), 6.99 (d, *J* = 7.1 Hz, 2H), 6.86 (d, *J* = 8.7 Hz, 1H), 6.48 (s, 1H), 6.27 (d, *J* = 15.5 Hz, 1H), 5.12 (s, 2H), 3.84 (s, 3H), 3.66 (s, 4H), 3.00 (s, 2H), 2.69 (s, 2H), 1.84 (s, 4H), and 1.30 (d, *J* = 3.1 Hz, 9H). ^13 ^C NMR (500 MHz, CDCl_3_): *δ* 167.52, 151.08, 150.70, 150.08, 149.64, 141.59, 137.66, 133.51, 128.49, 127.76, 127.23, 127.20, 125.58, 125.52, 123.85, 122.64, 121.92, 119.86, 117.91, 116.16, 113.34, 110.27, 70.75, 63.69, 55.99, 49.76, 40.72, 34.60, 31.34, 25.07, 23.01, and 22.66. HRMS (ESI) *m/z* calculated for C_36_H_41_N_3_O_3_ [M + H]^+^ 564.3221 was found to be 564.3216.

*(E)-3–(4-((4-cyanobenzyl)oxy)-3-methoxyphenyl)-N-(2-((1,2,3,4-tetrahydroacridin-9-yl)amino)ethyl)acrylamide (* ***10j*** *).* Yellow powder, yield: 22%, m.p. 88–90 °C. ^1^H NMR (300 MHz, CDCl_3_): *δ* 7.97 (d, *J* = 8.6 Hz, 1H), 7.87 (d, *J* = 8.2 Hz, 1H), 7.64 (t, *J* = 7.3 Hz, 2H), 7.54 (t, *J* = 7.5 Hz, 2H), 7.49–7.41 (m, 1H), 7.31–7.17 (m, 2H), 7.01 (d, *J* = 10.8 Hz, 2H), 6.76 (d, *J* = 8.2 Hz, 1H), 6.45 (d, *J* = 15.6 Hz, 1H), 5.19 (s, 2H), 3.87 (s, 3H), 3.73 (s, 4H), 2.98 (s, 2H), 2.63 (s, 2H), and 1.81 (s, 4H). ^13 ^C NMR (500 MHz, CDCl_3_): *δ* 168.02, 152.26, 149.76, 149.16, 142.15, 141.18, 132.48, 129.51, 128.70, 127.47, 125.22, 124.01, 123.29, 121.78, 118.76, 118.68, 118.25, 114.27, 113.61, 111.78, 110.52, 69.88, 56.03, 50.25, 40.28, 24.57, 22.62, and 21.92. HRMS (ESI) *m/z* calculated for C_33_H_32_N_4_O_3_ [M + H]^+^ 533.2547 was found to be 533.2542.

*(E)-3–(3-methoxy-4-((4-(trifluoromethyl)benzyl)oxy)phenyl)-N-(2-((1,2,3,4-tetrahydroacridin-9-yl)amino)ethyl)acrylamide (* ***10k*** *).* Yellow powder, yield: 51%, m.p. 60–62 °C. ^1^H NMR (300 MHz, CDCl_3_): *δ* 7.98 (d, *J* = 8.5 Hz, 1H), 7.90 (d, *J* = 8.4 Hz, 1H), 7.65 (d, *J* = 8.0 Hz, 2H), 7.59 (d, *J* = 7.7 Hz, 2H), 7.57–7.47 (m, 3H), 7.39–7.30 (m, 1H), 7.03 (d, *J* = 8.9 Hz, 2H), 6.84 (d, *J* = 8.0 Hz, 1H), 6.32 (s, 1H), 5.24 (s, 2H), 3.92 (s, 3H), 3.71 (s, 4H), 3.04 (s, 2H), 2.73 (s, 2H), and 1.88 (s, 4H). ^13 ^C NMR (500 MHz, CDCl_3_): *δ* 167.57, 158.03, 150.89, 149.68, 149.37, 141.27, 140.70, 128.58, 128.42, 127.89, 127.22, 125.61, 125.59, 125.56, 123.83, 122.74, 121.70, 119.71, 118.46, 115.91, 113.51, 110.44, 70.04, 55.96, 49.84, 40.70, 33.53, 25.02, 22.96, and 22.57. HRMS (ESI) *m/z* calculated for C_33_H_32_F_3_N_3_O_3_ [M + H]^+^ 576.2469 was found to be 576.2457.

*(E)-3–(3-methoxy-4-((3-methoxybenzyl)oxy)phenyl)-N-(2-((1,2,3,4-tetrahydroacridin-9-yl)amino)ethyl)acrylamide (* ***10l)***. Yellow powder, yield: 13%, m.p. 63–65 °C. ^1^H NMR (300 MHz, CDCl_3_): *δ* 7.96 (d, *J* = 8.4 Hz, 1H), 7.87 (d, *J* = 8.5 Hz, 1H), 7.58 (d, *J* = 15.5 Hz, 1H), 7.48 (t, *J* = 7.5 Hz, 1H), 7.29 (d, *J* = 8.2 Hz, 2H), 6.99 (d, *J* = 5.7 Hz, 4H), 6.83 (t, *J* = 7.0 Hz, 3H), 6.34 (d, *J* = 15.6 Hz, 1H), 5.15 (s, 2H), 3.86 (s, 3H), 3.80 (s, 3H), 3.69 (s, 4H), 2.99 (s, 2H), 2.66 (s, 2H), and 1.83 (s, 4H). ^13 ^C NMR (500 MHz, CDCl_3_): *δ* 168.59, 159.88, 153.53, 151.87, 149.97, 149.77, 141.64, 138.26, 130.43, 129.94, 129.90, 129.72, 127.95, 124.20, 123.72, 122.22, 119.36, 118.20, 113.49, 113.47, 112.68, 110.24, 70.75, 56.12, 55.27, 39.70, 29.34, 27.23, 22.29, and 21.29. HRMS (ESI) *m/z* calculated for C_33_H_35_N_3_O_4_ [M + H]^+^ 538.27 was found to be 538.2697.

*(E)-3–(3-methoxy-4-((4-methoxybenzyl)oxy)phenyl)-N-(2-((1,2,3,4-tetrahydroacridin-9-yl)amino)ethyl)acrylamide (* ***10m*** *).* Yellow powder, yield: 38%, m.p. 67–69 °C. ^1^H NMR (300 MHz, CDCl_3_): *δ* 8.06 (d, *J* = 8.3 Hz, 1H), 7.99 (d, *J* = 8.4 Hz, 1H), 7.66 (d, *J* = 15.4 Hz, 1H), 7.61 (d, *J* = 7.6 Hz, 1H), 7.43 (d, *J* = 8.8 Hz, 1H), 7.37 (t, *J* = 3.9 Hz, 2H), 7.10 (d, *J* = 3.7 Hz, 4H), 6.94 (d, *J* = 7.6 Hz, 2H), 6.36 (d, *J* = 15.6 Hz, 1H), 5.26 (s, 2H), 3.98 (s, 3H), 3.90 (s, 3H), 3.78 (s, 4H), 3.12 (s, 2H), 2.81 (s, 2H), and 1.96 (s, 4H). ^13 ^C NMR (500 MHz, CDCl_3_): *δ* 168.22, 159.84, 155.64, 152.26, 149.78, 149.63, 144.21, 141.26, 138.23, 129.70, 129.42, 128.06, 125.39, 123.95, 123.34, 121.87, 119.40, 118.43, 114.36, 113.43, 112.74, 110.40, 70.72, 56.00, 55.25, 50.27, 40.31, 32.00, 24.66, 22.65, and 21.97. HRMS (ESI) *m/z* calculated for C_33_H_35_N_3_O_4_ [M + H]^+^ 538.27 was found to be 538.2698.

### Biological activity

#### *In vitro* inhibitory evaluations on ChEs

AChE (EC 3.1.1.7, Type VI-S, from electric eel, C3389) and BuChE (EC 3.1.1.8, from equine serum, C0663), 5,5’-dithiobis (2-nitrobenzoic acid) (DTNB, D218200), acetylthiocholine iodide (ATC, A5751) and butyrylthiocholine iodide (BTC, B3253) used as substrates were obtained from Sigma-Aldrich (St. Louis, MO).

The inhibitory capacity of the synthesised compounds on AChE and BuChE biological activity in this paper were evaluated according to our previously reported method^21^. Briefly, AChE/BuChE stock solution was diluted with 0.2 M phosphate buffer pH 8.0 to give 2.5 units/mL (for electrophorus electricus (eeAChE) and equine serum (eqBuChE)). ATC/BTC iodide solution (0.075 M) was dissolved in deionized water. DTNB solution (0.01 M) was prepared using water containing 0.15% (w/v) sodium bicarbonate. The assay solution was prepared as follows: potassium dihydrogen phosphate (1.36 g, 10 mmol) was dissolved in 100 ml of water. The pH of the solution was then adjusted to 8.0 ± 0.1 with KOH. Stock solutions of the test samples were dissolved in ethanol to give a final concentration of 10 − 4^ ^M when diluted to the final volume of 3.32 ml. For each compound, a dilution series of five different concentrations 10 − 5–10 − 9^9 ^M were prepared.

For measurement, a cuvette containing 3 mL of phosphate buffer, 100 μL of eeAChE or eqBuChE, 100 μL of DTNB, and 100 μL of the test compound solution was added in sequence. The reaction was initiated by adding 20 μL of ATC or BTC and the solution was mixed immediately. Two minutes after ATC or BTC addition, the absorption was measured at 25 °C at 412 nm, using a Shimadzu 160 spectrophotometer. For the reference value, 100 μL of water was replaced for the test compound solution. For the blank control, additional 100 μL of water was also replaced for the enzyme solution. The measurement of each concentration was assayed in triplicate. GraphPad Prism 5 (GraphPad Software, Inc., La Jolla, CA) was used for data processing. The inhibition curve was fitted by plotting percentage enzyme activity (100% for the reference) versus the logarithm of test compound concentration. The half maximal inhibitory concentration (IC_50_) values were calculated according to the curve and the data were shown in mean ± SEM.

#### Inhibition of self-induced Aβ_1–42_ aggregation

Inhibitory effects of the compounds on self-induced Aβ_1–42_ aggregation were tested through a Thioflavin T (ThT)-(T3516, Sigma-Aldrich) binding assay. Firstly, aliquots of 2 μL of Aβ_1–42_ (AS-64129–05 Anaspec Inc.) containing 2 mg/mL HFIP (1,1,1,3,3,3-hexafluoro-2-propanol, 52517( Sigma-Aldrich) were stocked in DMSO. Then, they were diluted with 0.215 M sodium phosphate buffer (pH 8.0) to the final concentration of 500 μM. Test compounds were dissolved in DMSO and then prepared at a concentration of 25 μM by the buffer. Resveratrol was used as a positive control. The Aβ_1–42_ and the test sample solutions were incubated in a 96-well plate for 24 h at the room temperature. After the incubation, the tested compounds were diluted to a final volume of 150 μL with 50 mM glycine-NaOH buffer (pH 8.5) containing 5 mM ThT. Fluorescence intensity was read (excitation wavelength 450 nm, emission wavelength 485 nm) on a SpectraMax Paradigm Multimode Reader (Molecular Device, Sunnyvale, CA).

The calculation of the inhibitory rate of Aβ_1–42_ self-induced aggregation was performed as the following equation: (1 − *I_Fi_*/*I_Fc_*) × 100%. *I_Fi_* and *I_Fc_* were the fluorescence intensities measured in the presence and absence of inhibitors, respectively, after subtracting the background fluorescence of the 5 mM ThT solution. Each compound was measured in triplicate.

#### Behavioral testing

Behavioral studies were performed using the Morris water maze^25^ according to our method reported previously^26^. Adult male imprinting control region (ICR) mice (8–10 weeks old, weight 20–25 g) were obtained from the Yangzhou University Medical Center (Yangzhou, China). All animal handling and experimental protocols were approved by the Institutional Animal Care and Use Committee of the China Pharmaceutical University. Scopolamine hydrobromide was purchased from Aladdin Reagents (S107418, Shanghai, China). Tacrine that was used as the positive control was synthesized by our lab (purity >95%).

Thirty mice were randomly separated into five subgroups (six mice for each group): (i) vehicle as a blank control group, (ii) scopolamine as a model group, (iii) tacrine plus scopolamine as a positive control, (iv) compound **10d** plus scopolamine as a test group, and (v) compound **10g** plus scopolamine as a test group. The mice for model, tacrine, compounds **10d**, and **10g** groups received intraperitoneal (i.p). injections of scopolamine (1 mg/kg), while the blank control group was injected with saline. Tacrine, compounds **10d**, and **10g** were orally administered (30 mg/kg) to mice in groups (iii), (iv), and (v) 30 min before the i.p. administration of scopolamine or saline.

A circular pool (120 cm in diameter, 60 cm height) which was fixed with an escape platform (10 cm diameter) and filled to a depth of 40 cm fresh water (kept at 25 °C) composed the water maze and this water maze placed in a lit room was used to evaluate the cognition function and memory capacity of mice according to our previously described method^24^ . After five days of learning and memory training, a probe trial was performed on day 6. For the evaluation of cognitive function, each mouse was individually trained on a visible-platform (labeled by a small flag, 5 cm tall) for two days and was followed by a hidden-platform version (placed 1 cm below the surface of the water) of the water maze from day 3 to day 5. All mice were subjected to two training trials daily, each of which lasted for 90 s. The time for each mouse to find the platform (a successful escape) was recorded. If a mouse failed to reach the platform within 90 s, the test was terminated and the mouse was carefully navigated to the platform by hands. Each mouse was kept on the platform for 30 s whether it succeeded or failed to reach the platform. On the last day (day 6), a probe trial was given to the mice in which the platform was removed from the pool and each mouse was allowed 90 s to search for the platform. The time the animals spent to reach the missing platform location and the number of times they crossed that location were recorded.

Data for the time of escape latency, the swimming trajectory, and the number of visual platform crossings were recorded by Panlab SMART 3.0 (Panlab, S.L.U, Spain) and GraphPad Prism 5 was used to evaluate the level of memory retention.

#### Hepatotoxicity studies

Hepatotoxicity evaluation was performed on adult male ICR mice (8–10 weeks old, weighing 20–25 g) purchased from the Yangzhou University Medicine Centre (Yangzhou, China) according to our method previously described^26^. Tacrine, compounds **10d**, and **10g** were dissolved in a sodium carboxymethyl cellulose (CMC-Na) solution (0.5 g CMC-Na in 100 ml distilled water) corresponding to 3 mg/100 g body weight, concentration of 151.5 μmol/kg body weight were administered intragastrically (i.g.). Heparinised serum was obtained from the retrobulbar plexus 8, 22, and 36 h after the administration and were subjected to hepatotoxicity evaluation. The activity of aspartate aminotransferase (AST) and alanine aminotransferase (ALT), two indicators of liver damage, was determined by a commercial assay kit (EF551 and EF550 for ALT, EH027 and EF548 for AST, Wako, Japan). The data were analysed by Biochemical Analyzer (HITACHI 7020, Japan).

Mice were sacrificed 1 h after the collection of retrobulbar blood and livers were removed for morphological studies by using the immunohistochemical method. Two 3 mm sections of each liver extending from the hilus to the margin of the left lateral lobe were isolated using ultra-thin semiautomatic microtome (Leica RM2245, Germany) and placed in 10% buffered formaldehyde immediately, fixed for two days, and embedded together in one paraffin block by using paraffin embedding station (Leica EG1150H, Germany). Subsequently, 5 μm sections were prepared from these paraffin blocks. They were deparaffinated and stained with hematoxylin and eosin (H&E) for histopathological examinations.

### Molecular docking studies

Molecular docking studies were completed by CDOCKER module implemented in Discovery Studio (DS; version 3.0, BIOVIA, San Diego, CA). The X-ray crystal structures of huAChE and huBuChE with small molecular ligands were downloaded from protein data bank (PDB, ID: 4EY7, 4TPK)^27^ ^,^ ^28^. The structures were initially processed by “prepare protein” module in DS to give the structures suitable for docking. Missed sidechains of the proteins were added and the water molecules were removed, later the structures were protonated at pH 7.4. “Prepare ligands” module in DS was applied for the structural preparation of the test compounds, which were then protonated at pH 7.4. The resulted molecules were subsequently minimized by “minimize ligands” module. The “full minimisation” algorithm was used to carry out the minimisation, with max steps set to 2000, the root mean square (RMS) gradient set to 0.01. Other parameters were set as default.

For molecular docking, the binding site was defined as a site sphere (in 10 Å radius) around the original ligands in the co-crystal structures. The simulated annealing parameters were set as follows: heating steps and cooling steps were set to 2000 and 5000, respectively, while heating and cooling temperature was set to 700 and 300, respectively. Other parameters were kept as default. Ten top-ranked conformations for each docked compound were retained and visually inspected for binding pattern analysis, which was visualised and depicted in PyMOL^29^ software.

## Results

### Compound design and chemistry

Compound **10a**, which was previously reported by our group, was used as the lead compound for structural modification^24^. Differently substituted benzyl was introduced to the ferulic acid moiety of **10a** (Table 1).

**Table 1.** Structures, eeAChE and eqBuChE inhibitory activities of target compounds.


Compound	R	IC_50_ (nM) ± SEM^a^		SI^d^
		AChE^b^	BuChE^c^	
**10a**	H	92.2 ± 18.3	215.4 ± 39.5	0.4
**10b**	4-F	114.8 ± 31.2	163.1 ± 16.6	0.7
**10c**	4-Cl	100.8 ± 14.6	62.5 ± 9.9	1.6
**10d**	4-Br	49.5 ± 3.8	69.4 ± 13.4	0.7
**10e**	2-CH_3_	64.5 ± 7.4	98.6 ± 19.1	0.7
**10f**	3-CH_3_	63.2 ± 13.5	256.7 ± 3.7	0.2
**10g**	3,4-diCH_3_	37.0 ± 6.4	101.4 ± 15.5	0.4
**10h**	4-CH_3_	139.9 ± 34.4	147.5 ± 22.4	0.9
**10i**	4-*t*-Bu	284.1 ± 59.2	158.3 ± 22.8	1.8
**10j**	4-CN	107.1 ± 29.9	79.8 ± 8.9	1.3
**10k**	4-CF_3_	128.6 ± 16.5	52.7 ± 6.3	2.4
**10l**	3-OCH_3_	143.0 ± 40.1	195.2 ± 16.5	0.7
**10m**	4-OCH_3_	75.5 ± 12.8	134.0 ± 11.1	0.6
Tacrine	–	14.5 ± 2.6	4.5 ± 0.4	3.2

a Concentration required for 50% inhibition of ChEs, data were shown in mean ± SEM of triplicate independent experiments.

b AChE (EC 3.1.1.7) from electric eel.

c BuChE (EC 3.1.1.8) from horse serum.

d Selectivity index (SI) is defined as AChE IC_50_/BuChE IC_50_.

**Scheme 1.**
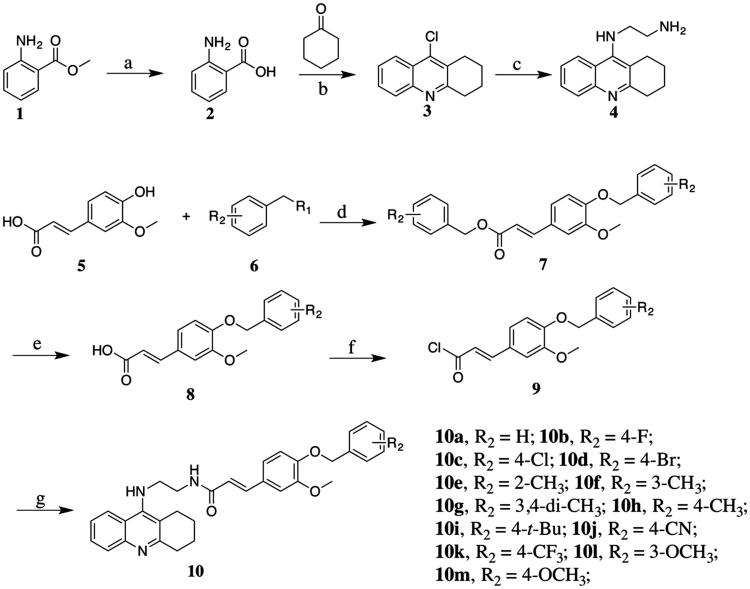
Reagents and conditions: (a) NaOH, H_2_O, room temperature (r.t), 12 h; (b) POCl_3_, reflux, 3 h; (c) 1-pentanol, ethylenediamine, 160 °C, 18 h; (d) K_2_CO_3_, DMF, 82 °C, 4 h; (e) NaOH, MeOH, H_2_O, 82 °C, 3 h; (f) SOCl_2_, CH_2_Cl_2_, reflux, 3 h; and (g) K_2_CO_3_, Na_2_SO_4_, CH_2_Cl_2_, r.t, 24 h.

The preparation of the target compounds started from methyl 2-aminobenzoate^1^ through seven steps as described in Scheme 1 methyl 2-aminobenzoate^1^ was hydrolysed to yield 2-aminobenzoic acid^2^, which is condensed with cyclohexanone to give 9-chloro-1,2,3,4-tetrahydroacridine^3^. N^1^–(1,2,3,4-tetrahydroacridin-9-yl)ethane-1,2-diamine^4^ was obtained by aminolysis of 9-chloro-1,2,3,4-tetrahydroacridine with ethane-1,2-diamine. Compound **5** was substituted with benzyl bromide or benzyl chloride, followed by the hydrolysis of ester group to form compound **8a**–**8m**. Then, the hydrolysates were treated with thionyl chloride to prepare the corresponding chlorides, which were condensed with compound **4** to afford target compounds **10a**–**10m**.

### Cholinesterase inhibitory activity and SAR analysis

All the synthesised compounds were assayed against AChE from eeAChE and BuChE from eqBuChE according to the modified method of Ellman et al.^30^, using tacrine as a reference standard. Inhibitory activities were presented as IC_50_ (nM). The activity results and selectivity index (SI) are summarized in Table 1. All the compounds displayed potent inhibitory activities, with IC_50_ values in the two or three-digit nanomolar range.

Firstly, we introduced methyl group to the benzyloxyl moiety. Compared to compound **10a**, methyl substitution at *ortho*- or *meta*- position of benzyloxyl (**10e** and **10f**, respectively) showed enhanced activities while the *para*-substituted compound led to reduced inhibitory activity and lower selectivity against AChE. Interestingly, for the multi-substituted compound, the 3,4-diCH_3_ (**10g**) was the most potent AChE inhibitor (IC_50_ = 37.02 nM).

Next, we explored the inductive effects of substituents on ChEs inhibitory activities. The electron-withdrawing groups (EWGs), such as −CN (**10j**) and −CF_3_ (**10k**) showed reduced AChE inhibitory activity while they were favorable for BuChE inhibition.

Then, we designed compounds bearing halogen substitution. 4-F (**10b**) and 4-Cl (**10c**) showed comparable activity to compound **10a**, but the 4-Br substituted compound (**10d**) displayed obvious improvement. The impact was consistent with the results mentioned above. The stronger electron-withdrawing ability of the substituent was, the poorer the inhibition activity of the compound was.

Next, we synthesised compounds with electron-donating groups. *para*-substituted –OCH_3_ (**10m**) showed comparable activity to **10a**. However, replacement with 3-OCH_3_ (**10l**) and 4-*t*-Bu (**10i**) was not tolerated.

### Binding mode analysis by molecular modeling

**Figure 1.**
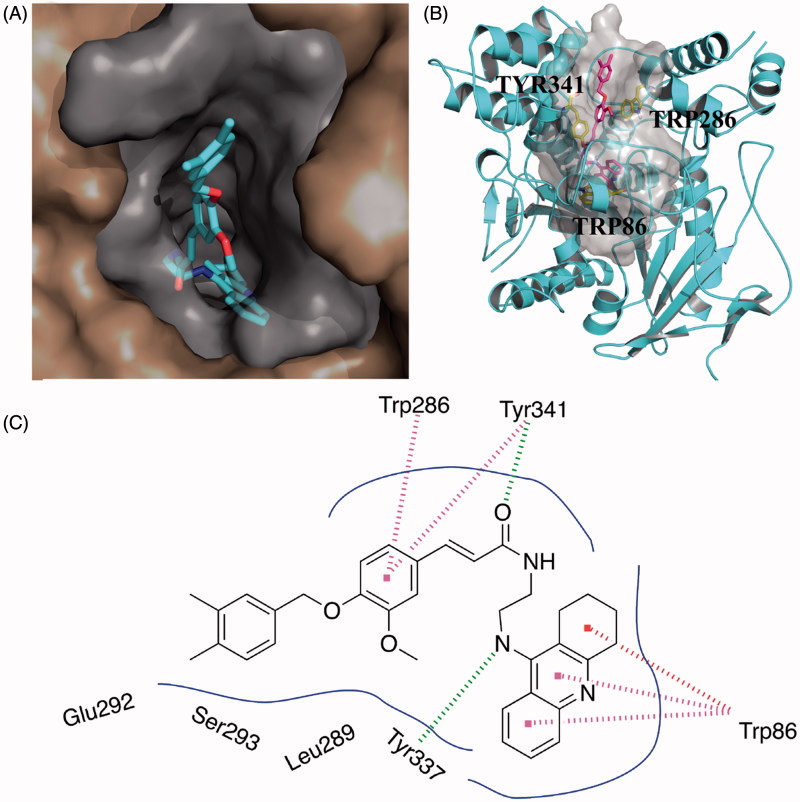
Predicted binding mode for Compound **10g** with AChE. (A) View from the top of the binding gorge, showing Compound **10g** as cyan sticks, bound to the surface of huAChE (PDB code: 4EY7). (B) Profile view of Compound **10g** as magenta sticks bound in the gorge pocket of huAChE (grey surface). Key residues for the binding of Compound **10g** are shown as yellow sticks. (C) Two dimensional schematic diagram of docking model of Compound **10g** with AChE. Intermolecular interactions were described as dashed lines in different colors: green: hydrogen bond; purple: π–π stacking; and red: π–π T-shaped.

To further explore the binding interactions of the synthesised compounds with ChEs, we next carried out molecular docking studies by using DS. Binding mode of huAChE-**10g** and huBuChE-**10k** were analysed. The two compounds were selected as representatives for their most potent activity on AChE or BuChE. As shown in Figure 1, compound **10g** simultaneously occupied both the CAS and PAS of AChE. The 1,2,3,4-tetrahydroacridin core was located at the CAS through a π-π stacking interaction with Trp86. The NH- group on 1,2,3,4-tetrahydroacridin ring interacted with the backbone of Tyr337 through a hydrogen bond. The carbonyl group of the ferulic acid moiety formed a hydrogen bond with the side chain of Tyr341. The phenyl core of the ferulic acid moiety formed π-π stacking interactions with Trp286 and Tyr341. Such polar interactions stabilised the occupation of the ferulic acid moiety in the PAS site. The phenyl rings interacted with multiple residues through van der Waals interactions, such as Leu289, Ser293, and Tyr124.

**Figure 2.**
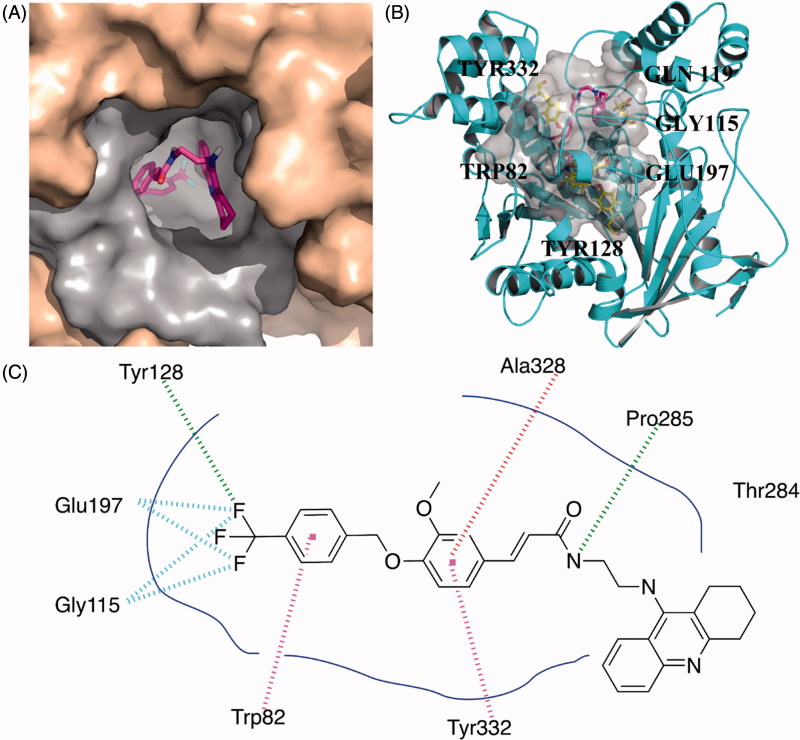
Predicted binding mode for Compound **10k** with BuChE. (A) View from the top of the binding cave, showing Compound **10k** as magenta sticks, bound to the surface of huBuChE (PDB code: 4TPK). (B) Profile view of Compound **10k** as magenta sticks bound in the pocket of huBuChE (grey surface). Key residues for the binding of Compound **10k** are shown as yellow sticks. (C) Two dimensional schematic diagram of docking model of Compound **10k** with BuChE. Intermolecular interactions were described as dashed lines in different colors: green: hydrogen bond; purple: π–π stacking; red: π-alkyl contact.

Compared to the narrow and long binding site of huAChE, huBuChE contains a much broader and larger binding cavity. As a result, compound **10k** exhibited a U-shaped conformation (Figure 2), which was obviously different from the linear conformation of **10g**. Therefore, the U-shaped conformation of **10k** can better occupy the active site of BuChE. For benzyloxyl moiety, the phenyl ring interacted with Trp 82 through π–π stacking. The fluorine atoms formed a hydrogen bond with Tyr128 and two fluorine bonds with Gly115 and Glu197, which better occupied the bottom of the binding pocket. The phenyl ring of the ferulic acid interacted with Tyr332 and Ala328 through π–π stacking and π-Alkyl contact, respectively. The nitrogen atom of amide moiety formed a hydrogen bond with Pro285. Multiple van der Waals contacts were observed between compound **10k** and different residues such as Thr284 and Ala328, providing stronger binding affinity.

### Inhibition of self-induced Aβ_1–42_ aggregation by selected compounds

The formation and accumulation of Aβ_1–42_ in the brain lead to neurotoxicity in AD^30^. Thus, targeting self-induced Aβ_1–42_ aggregation represents a promising strategy to discover novel neuroprotective agents^31^. Furthermore, one single molecule simultaneously inhibits Aβ_1–42_ aggregation and ChEs activity resulting in an anti-AD agent that is beneficial in the early and advanced stages of AD^32^ ^,^ ^33^.

Compounds **10d**, **10 g**, and **10j** that showed potent ChEs inhibition activity were selected for further evaluation for their inhibitory capacity on self-induced Aβ_1–42_ aggregation. A thioflavin T-based fluorometric assay was performed^34^. Results are summarised in Table 2. Three compounds displayed significant inhibition on the aggregation of Aβ_1–42_ with the inhibitory rate 66.84, 65.49, and 49.51%, respectively compared to 45.72% with resveratrol as reference standard under the concentration of 25 μM. These results suggested that our compounds were MTDLs. Because of these promising *in vitro* data, the *in vivo* activities of compounds **10d** and **10g** were further tested.

**Table 2.** Inhibition of Aβ_1–42_ self-induced aggregation of the synthesised compounds.

Compound	Inhibitory rate^a^ of compounds (25 μM) on self-induced Aβ_1-42_ aggregation (%)
**10d**	66.84
**10g**	65.49
**10j**	49.51
Resveratrol	45.72

a Inhibitory rate = (1 − *I_Fi_*/*I_Fc_*) × 100%, *I_Fi_* and *I_Fc_* were the fluorescence measured in the presence and absence of the tested compound.

### Behavioral studies

**Figure 3.**
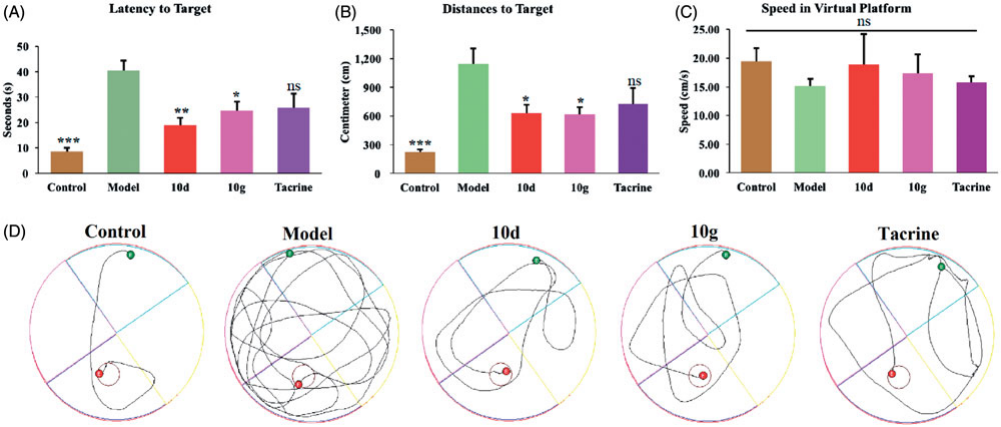
Effects of memory retention determined by the Morris water maze test. (A) The escape latency values to target. (B) The swimming distance to target. (C) The swimming speed in virtual platform. (D) The representative tracks of mice in each group in the Morris water maze test. Data are presented as the mean ± SEM (*n* = 6; ns *p* > 0.05, *p < 0.05, **p < 0.01, and ***p < 0.0001 versus the model group).

To further evaluate the ability of the synthesised compound to improve the cognitive ability, the optimal compounds **10d** and **10g**, were selected for *in vivo* behavioral analysis in scopolamine-induced cognition-impaired adult ICR mice (male mice, 8–10 weeks old, weight 20–25 g) using a Morris water maze with tacrine at 30 mg/kg as a positive control. Thirty mice were randomly allocated into five subgroups (*n* = 6 for each group): control, model, tacrine, compounds **10d**, and **10g**. The mean escape latency values, searching distances and swimming speed of all the groups are shown in Table 3. It was clear that administration of scopolamine led to a remarkable delay of the escape latency (40.51 ± 3.83 vs. 8.61 ± 1.53 s, *p* < 0.001) as compared to the control group, which suggested that administration of scopolamine led to a spatial memory deficiency in the mice. The time of escape latency for mice that were administered tacrine (25.85 ± 5.52 s, *p* > 0.05), compounds **10d** (19.08 ± 2.56 s, *p* < 0.01), and **10g** (24.73 ± 3.54 s, *p* < 0.05) were remarkably reduced compared to the model group. Interestingly, the mice treated with compounds **10d** (19.08 ± 2.56 s, *p* < 0.01), and **10g** (24.73 ± 3.54 s, *p* < 0.05) both demonstrated a more favorable amelioration of the cognitive and memory functions compared to the tacrine (25.85 ± 5.52 s, *p* > 0.05) group (Figure 3(A)). Similarity, tacrine group (725.64 ± 165.15 cm, *p* > 0.05), compounds **10d** group (628.95 ± 87.55 cm, *p* < 0.05), and **10g** group (612.85 ± 81.54 cm, *p* < 0.05) demonstrated a significantly shortened distance to the target than the model group (1146.00 ± 158.78 cm). Besides, the distance to the target for compounds **10d** (628.95 ± 87.55 cm, *p* < 0.05) and **10g** (612.85 ± 81.54 cm, *p* < 0.05) group were obviously shorter than tacrine group (725.64 ± 165.15 cm, *p* > 0.05), which further suggested that compounds **10d** and **10 g** were more favorable (Figure 3(B)). The results presented in Figure 3(C) showed that the swimming speed for each group was almost equivalent, which demonstrated that long-term use of compounds **10d** and **10g** are safe at 30 mg/kg/day. Besides, compounds **10d** (18.82 ± 5.36 cm/s) and **10 g** (17.27 ± 3.39 cm/s) did not injure the moving and exploring activities of mice, which are safer than tacrine (15.78 ± 1.03 cm/s). Meanwhile, the data of escape latency and searching distance were also supported by the analysis of the swimming trajectories of the mice in each group. For the mice in scopolamine model group (Figure 3(D)), the trajectory was longer and more disordered than the control group. The performance of tacrine group was slightly improved. Mice treated with compounds **10d** and **10g** showed much-shortened distances compared to tacrine group. Taken together, these behavioral performances demonstrated that compounds **10d** and **10g** can markedly improve the cognitive function of mice. The effectivity of **10d** and **10g** for symptom amelioration in AD mice model identifies them as potential anti-AD agents, especially for the treatment of cholinergic deficiency.

**Table 3.** The mean escape latency, searching distances, and swimming speed of five subgroups.

Group	Latency to target (s)^a^	Distance to target (cm)	Speed in virtual platform (cm/s)
Control	8.61 ± 1.53	218.50 ± 30.09	19.42 ± 2.30
Model	40.51 ± 3.83	1146.00 ± 158.78	15.16 ± 1.18
Compound **10d**	19.08 ± 2.56	628.95 ± 87.55	18.82 ± 5.36
Compound **10g**	24.73 ± 3.54	612.85 ± 81.54	17.27 ± 3.39
Tacrine	25.85 ± 5.52	725.64 ± 165.15	15.78 ± 1.03

a Values are expressed as the mean ± SEM of six independent experiments.

### Hepatotoxicity studies

**Figure 4.**
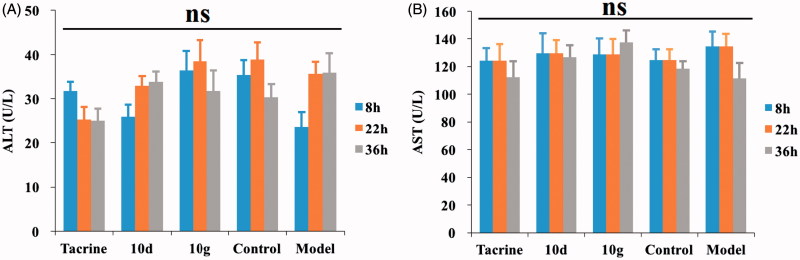
The ALT and AST levels. (A) The ALT levels of five subgroups. (B) The AST levels of five subgroups. Data are presented as the mean ± SEM (*n* = 6; ns *p* > 0.05).

**Figure 5.**
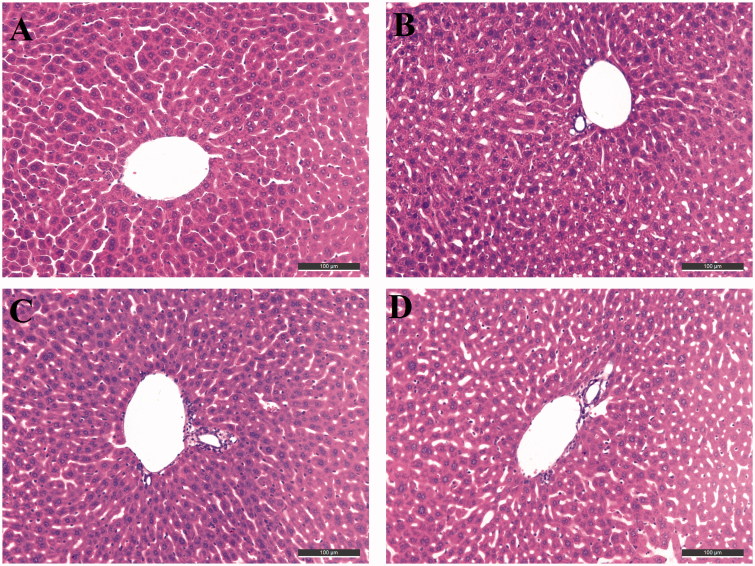
Histopathological study of livers of male mice after treatment with the solvent only (control, A), or 36 h after administration of Compound **10d** (B), Compound **10g** (C), and tacrine (D). HE staining, original magnification ×200.

To determine the possible drug-induced hepatotoxicity, **10d** and **10g** were selected for preliminary hepatotoxicity evaluation. The ALT and AST levels were measured, as shown in Table 4 and Figure 4. Heparinised serum was obtained after the treatment of the compounds at 8, 22, and 36 h, respectively. The levels of ALT and AST were comparable to those from the control group and model group (*p* > 0.05) at the three-time points, which suggested that compounds **10d** and **10g** had preliminary safety. Additionally, immunohistochemical staining was performed on the liver tissues for the histopathological study. The morphologic results of our tested compounds **10d** and **10g** were in accordance with the ALT and AST data. Treatment of compounds **10d** (Figure 5(B)) and **10g** (Figure 5(C)) did not cause adverse morphologic changes in liver compared to the control group (Figure 5(A)). However, when we investigated the antiproliferative activity of compounds **10d** and **10g** on HepG2 cells by 3-(4,5-dimethylthiazol-2-yl)-2,5-diphenyltetrazolium bromide (MTT) assays, we surprisingly observed that the two compounds showed relatively strong toxic effects (Supplementary Table S1). The two compounds showed high antiproliferative potency at 5 μM, however, we did not observe obvious hepatoxicity in the *in vivo* assays, even at a much higher concentration. In summary, the results indicated that these compounds may still have potential hepatoxicity especially in long-term use, but considering the much lower IC_50_ values of the two compounds on ChEs compared to the MTT assays, such potential hepatoxicity can be controlled by rational administration of the molecules. Additionally, the results also inspired us to further improve the safety of compounds in the subsequent structural modification of these hybrids.

**Table 4.** The ALT and AST levels (U/L) at the three-time points of five subgroups.

Group	ALT (U/L)^a^		
	8 h	22 h	36 h
Control	35.4 ± 3.4	39.0 ± 3.8	30.4 ± 3.0
Model	23.7 ± 3.3	35.7 ± 2.6	35.9 ± 4.4
Tacrine	31.8 ± 2.0	25.4 ± 2.8	25.1 ± 2.7
Compound **10d**	26.0 ± 2.7	32.9 ± 2.3	33.9 ± 2.3
Compound **10g**	36.4 ± 4.4	38.5 ± 4.8	31.8 ± 4.7
	AST (U/L)		
Group	8 h	22 h	36 h
Control	118.9 ± 8.1	124.6 ± 8.0	118.3 ± 5.6
Model	114.1 ± 10.8	134.6 ± 9.0	111.4 ± 11.4
Tacrine	133.7 ± 9.0	124.3 ± 12.1	112.4 ± 11.4
Compound **10d**	130.9 ± 14.5	129.6 ± 9.5	126.8 ± 8.7
Compound **10g**	122.1 ± 11.6	128.7 ± 11.2	137.3 ± 9.0

a Values are expressed as the mean ± SEM of six independent experiments.

## Conclusion

In summary, our study involved the design, synthesis and biological evaluation of a series of tacrine-ferulic acid hybrids with the aim to identify MTDLs for the treatment of AD. All of the target compounds simultaneously inhibited AChE and BuChE with the IC_50_ values in the nanomolar range. The binding manner of compound **10g** to huAChE and compound **10k** to huBuChE were also analysed by molecular docking studies. The representatives, compounds **10d**, **10g**, and **10j**, significantly inhibited the Aβ_1–42_ self-induced aggregation. In subsequent *in vivo* evaluation, the most promising compound **10 g** showed a remarkable improvement of memory in the scopolamine-induced cognitive impairment in the Morris water maze test. Additionally, compound **10g** exhibited preliminary safety in *in vivo* hepatotoxicity assays, without improving the level of ALT and AST. Overall, our findings provide a valuable lead compound for further development of novel multifunctional agents for Alzheimer’s disease.

## Supplementary Material

IENZ_1430691_Supplementary_Material.pdf
