# Supplementary material for: Synthesis, pharmacology and molecular docking on multifunctional tacrine-ferulic acid hybrids as cholinesterase inhibitors against Alzheimer’s disease
Source: J Enzyme Inhib Med Chem. 2018 Feb 6;33(1):496–506. doi: 10.1080/14756366.2018.1430691 (PMC6010002; doi:10.1080/14756366.2018.1430691)
Supplement: IENZ_1430691_Supplementary_Material.pdf [file IENZ_A_1430691_SM5110.pdf]

Supporting Information

Synthesis, Pharmacology and Molecular Docking on Multifunctional Tacrine-Ferulic Acid Hybrids as Cholinesterase Inhibitors against Alzheimer's Disease

Jie Zhu [a], Hongyu Yang [a], Yao Chen [b], Hongzhi Lin [a], Qi Li [a], Jun Mo [a], Yaoyao Bian [c], Yuqiong Pei [b], Haopeng Sun [a*]

[a] *Department of Medicinal Chemistry, China Pharmaceutical University, Nanjing, 210009, China;*

[b] *School of Pharmacy, Nanjing University of Chinese Medicine, Nanjing, 210023, China;*

[c] *School of Nursing, Nanjing University of Chinese Medicine, Nanjing, 210023, China;*

Corresponding author

Haopeng Sun: Tel: +86-25-85863169, E-mail: sunhaopeng@163.com;

The ^{1}H-NMR and ^{13}C-NMR spectrum of target compounds

10a ^{1}H-NMR

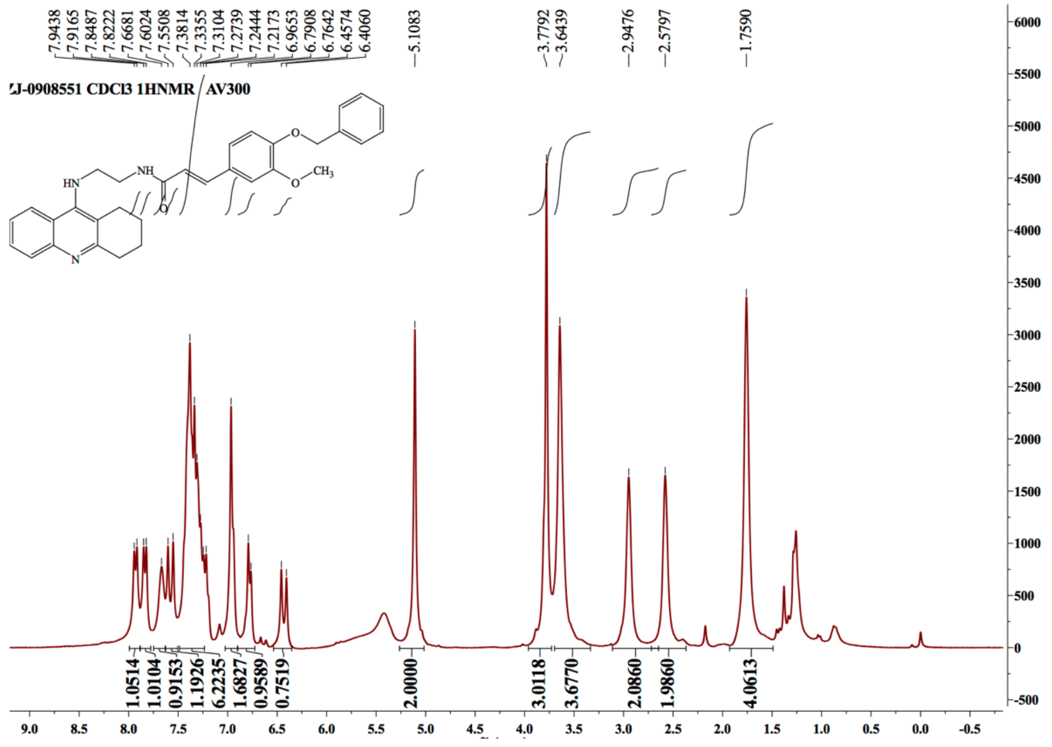

10a ^{13}C-NMR

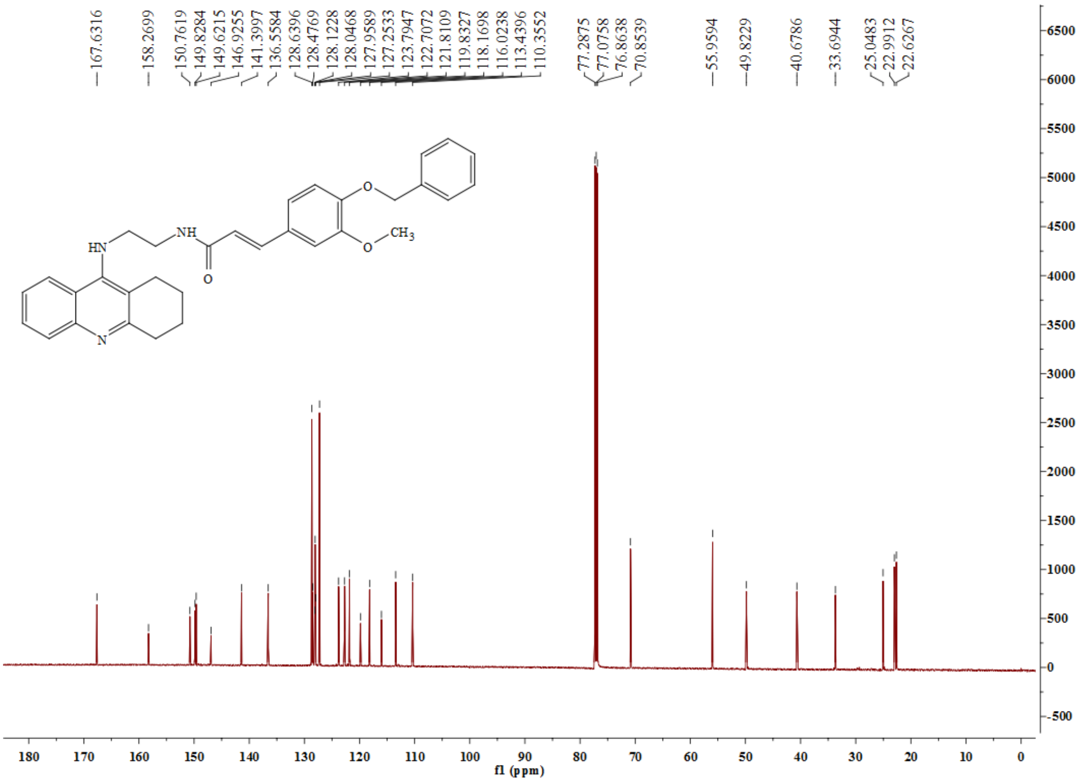

10b ^{1}H-NMR

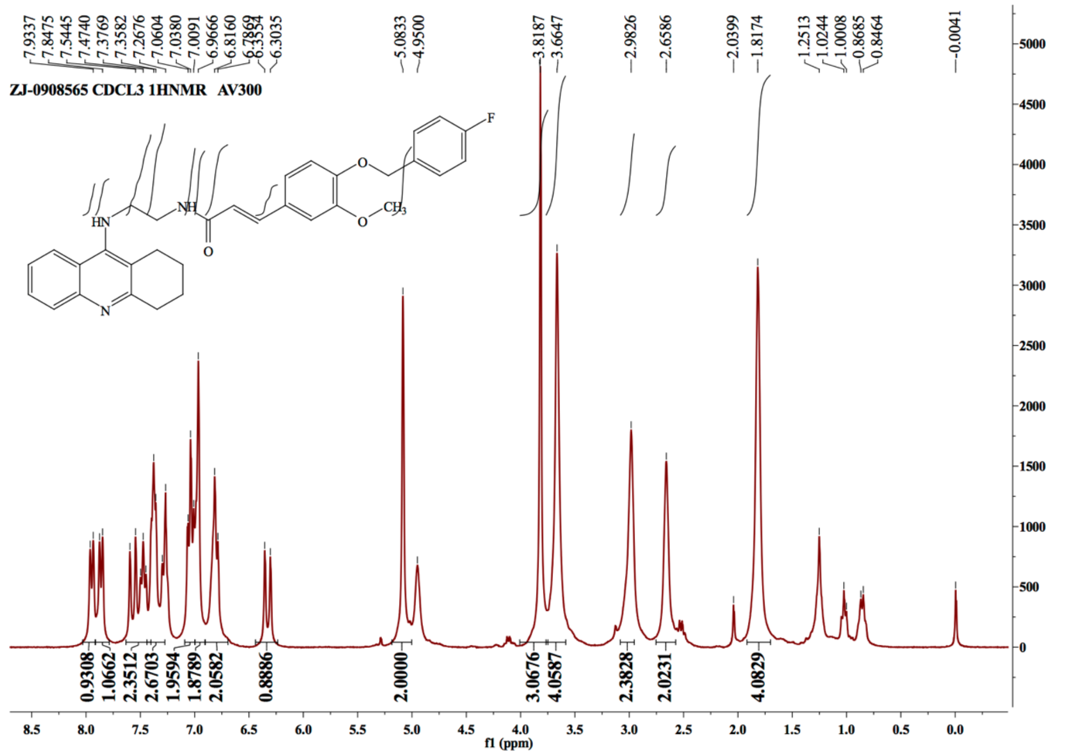

10b ^{13}C-NMR

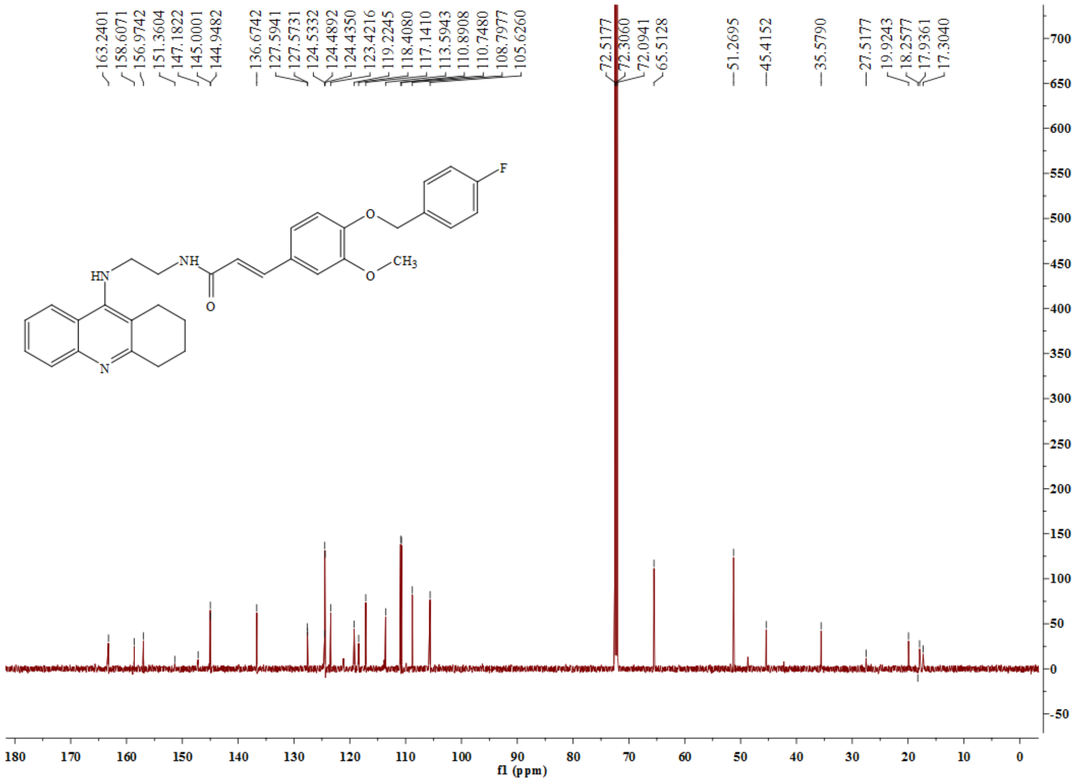

10c ^{1}H-NMR

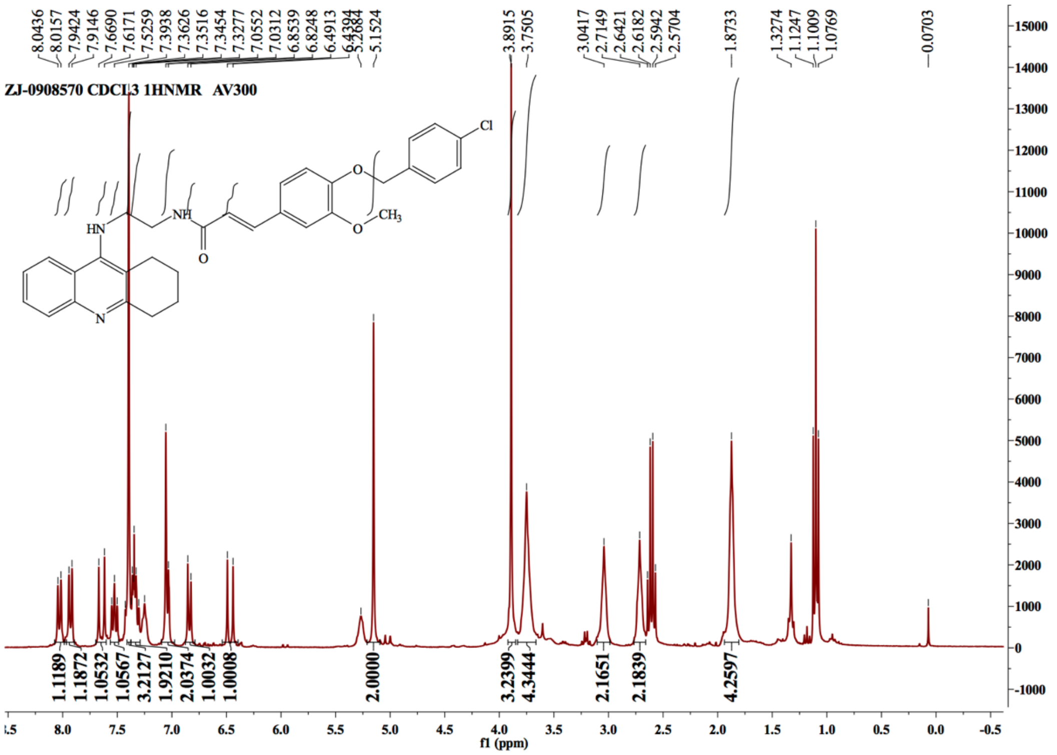

10c ^{13}C-NMR

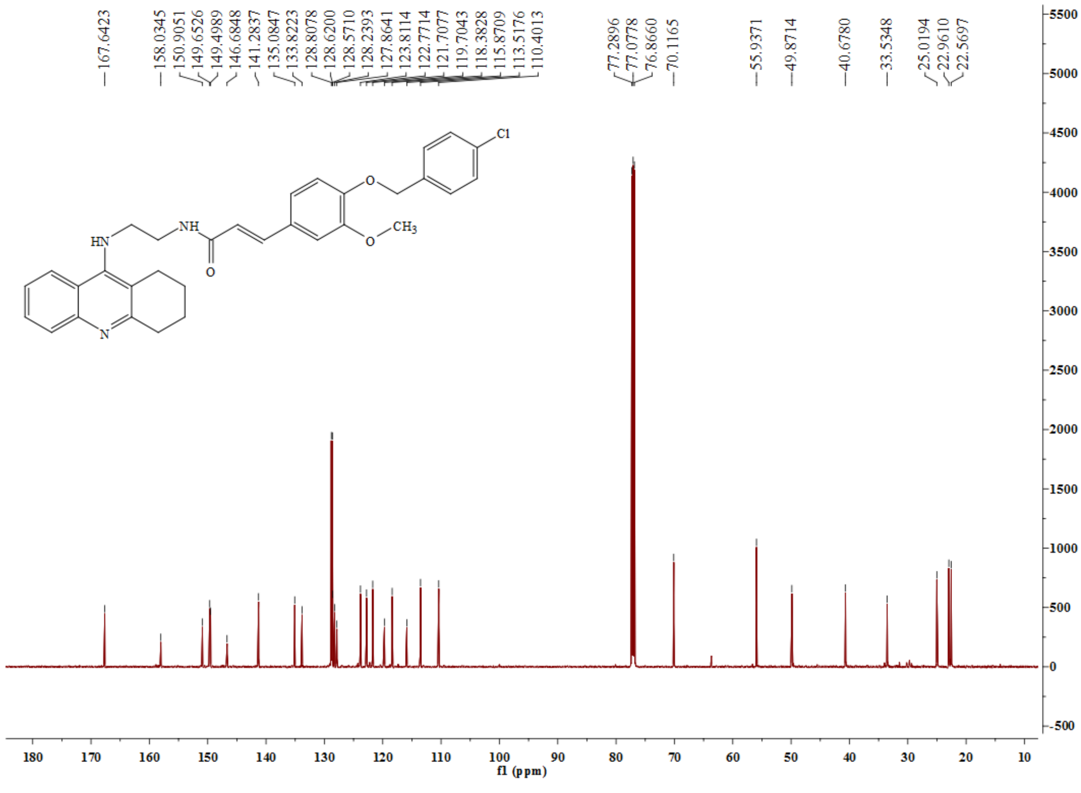

10d 1H-NMR

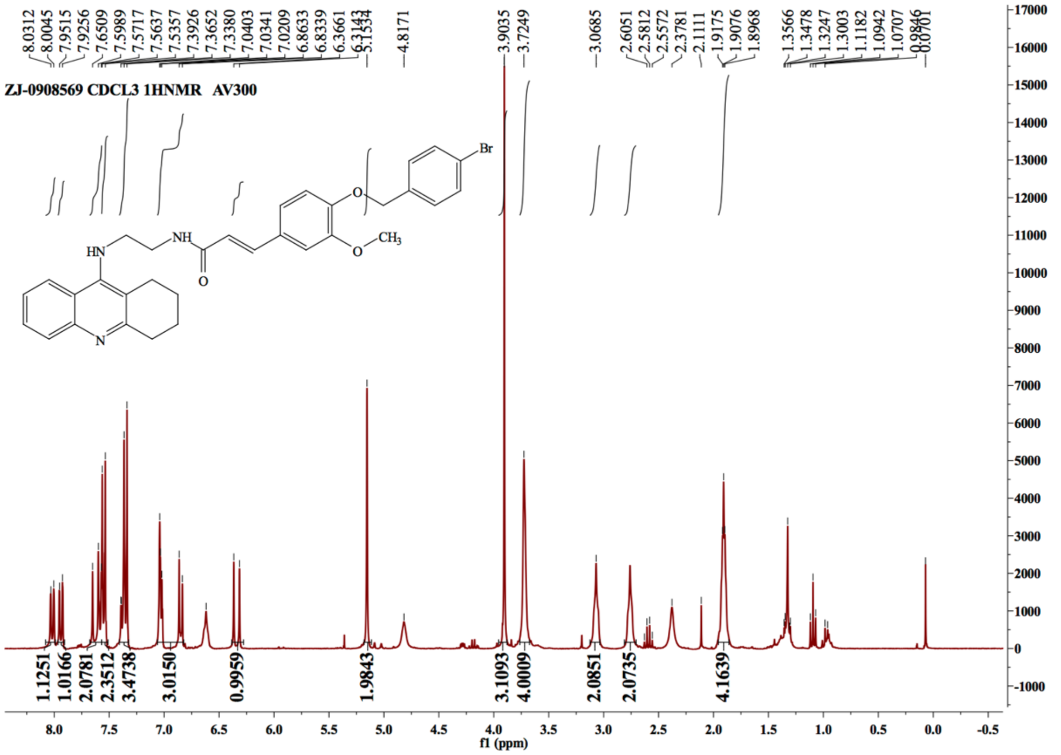

10d 13C-NMR

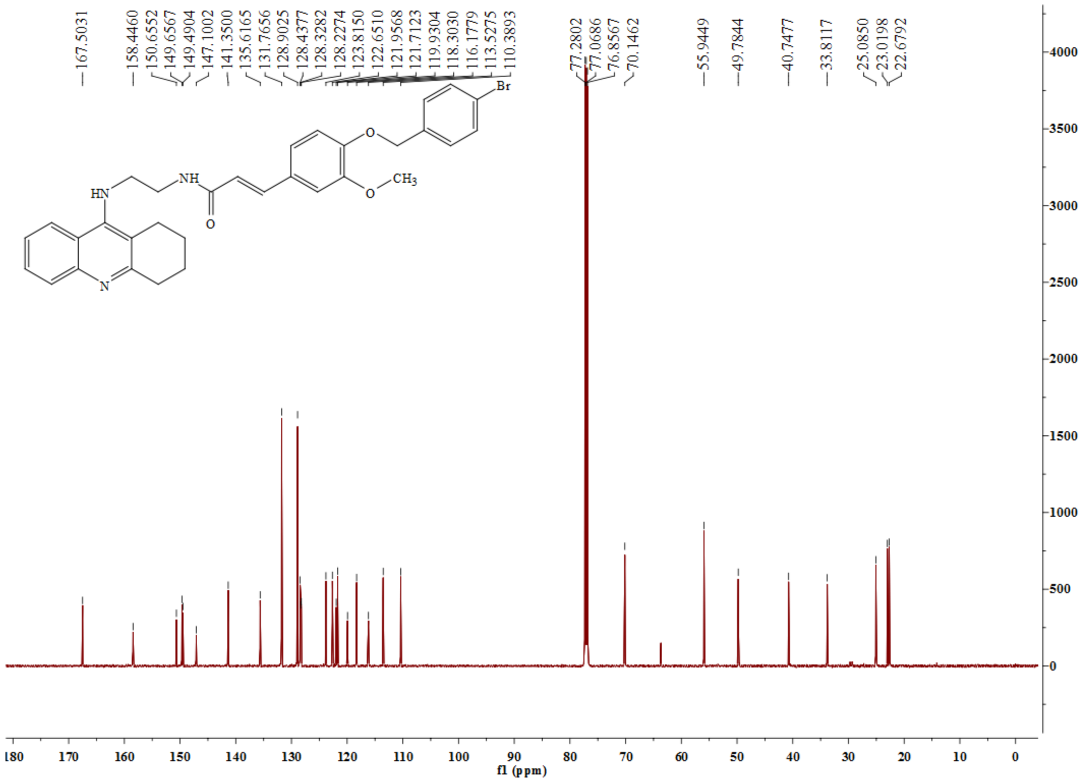

10e ^{1}H-NMR

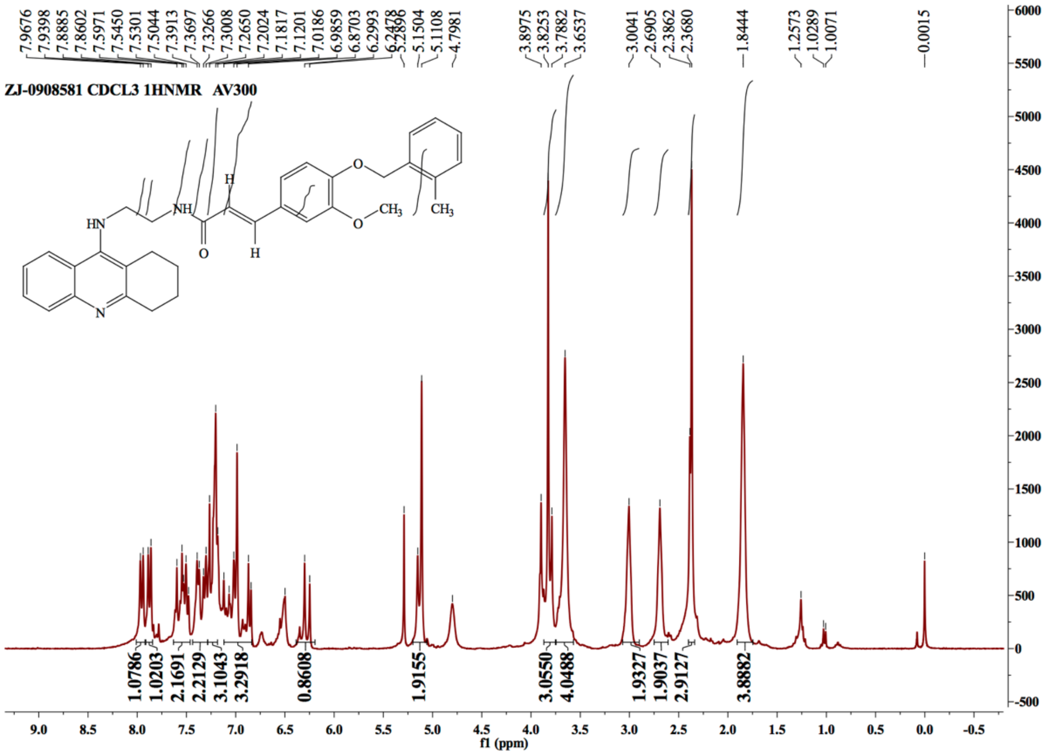

10e ^{13}C-NMR

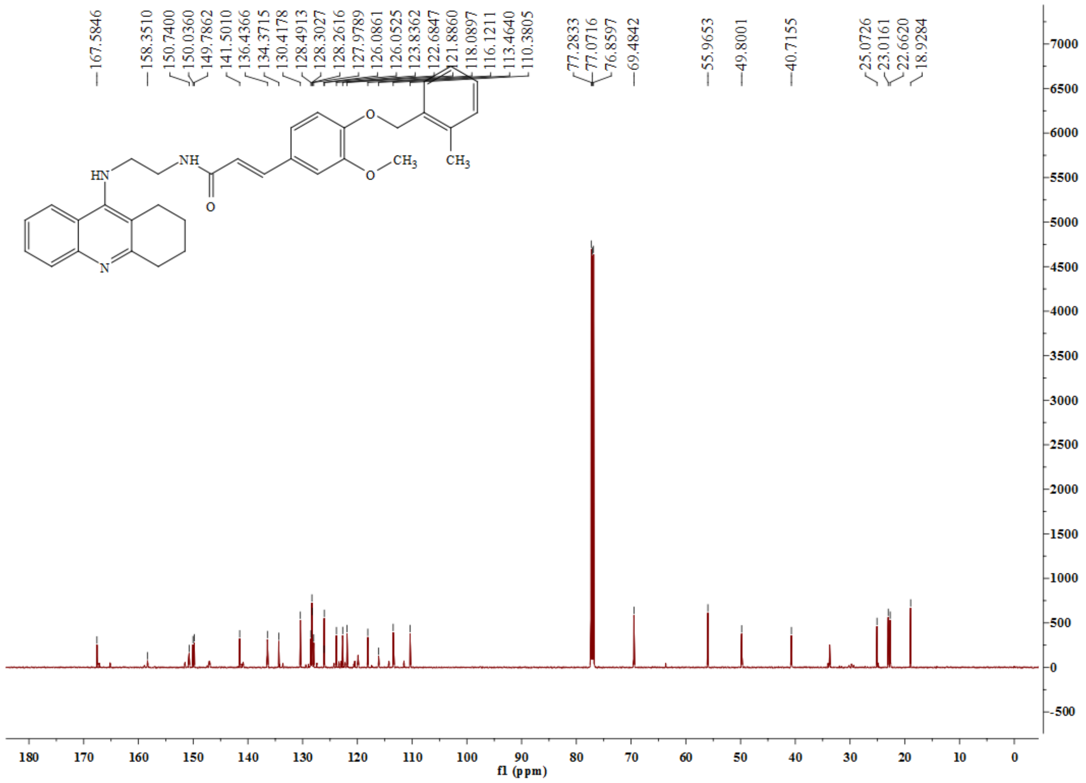

10f ^{1}H-NMR

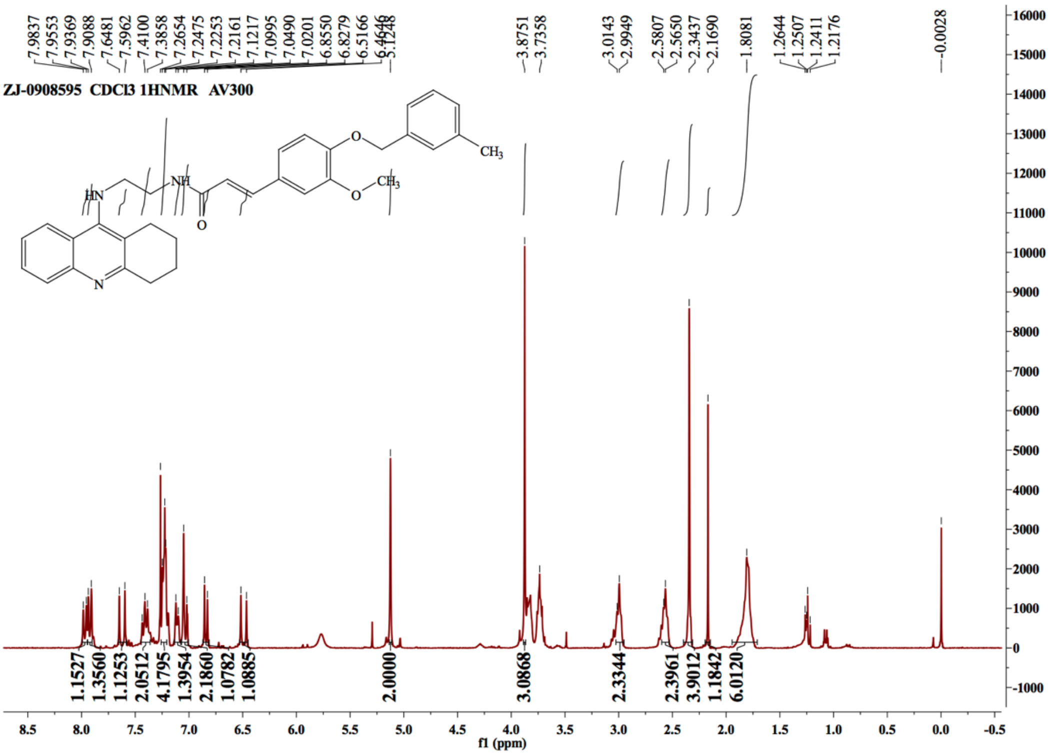

10f ^{13}C-NMR

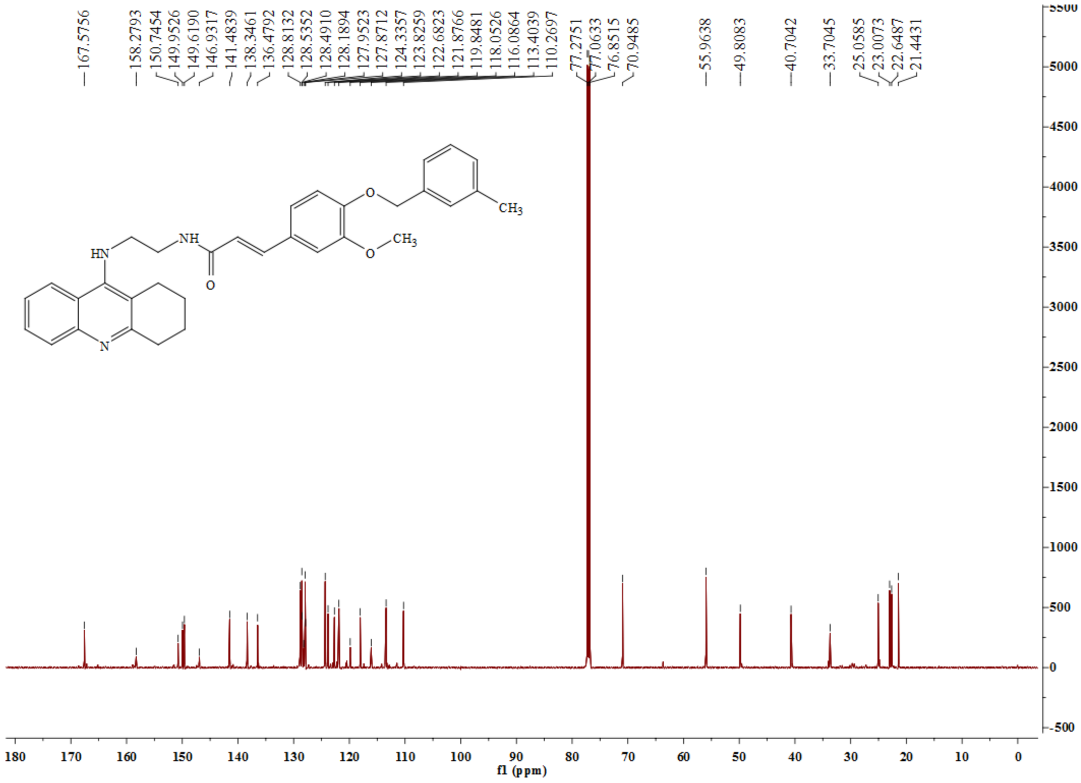

10g ^{1}H-NMR

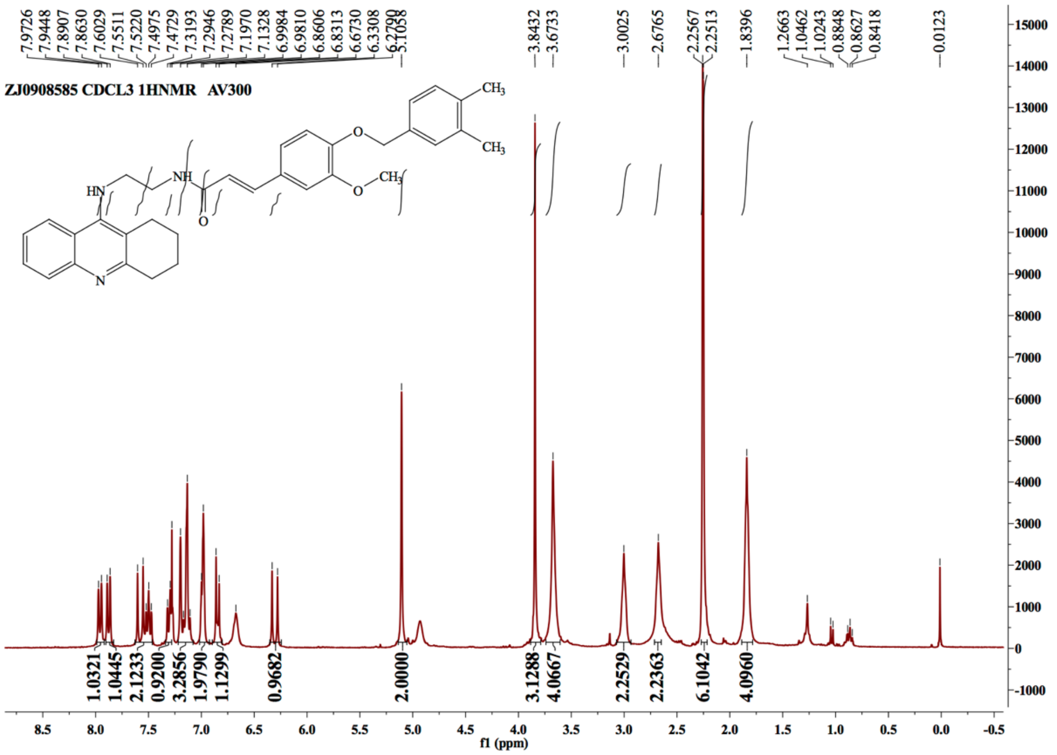

10g ^{13}C-NMR

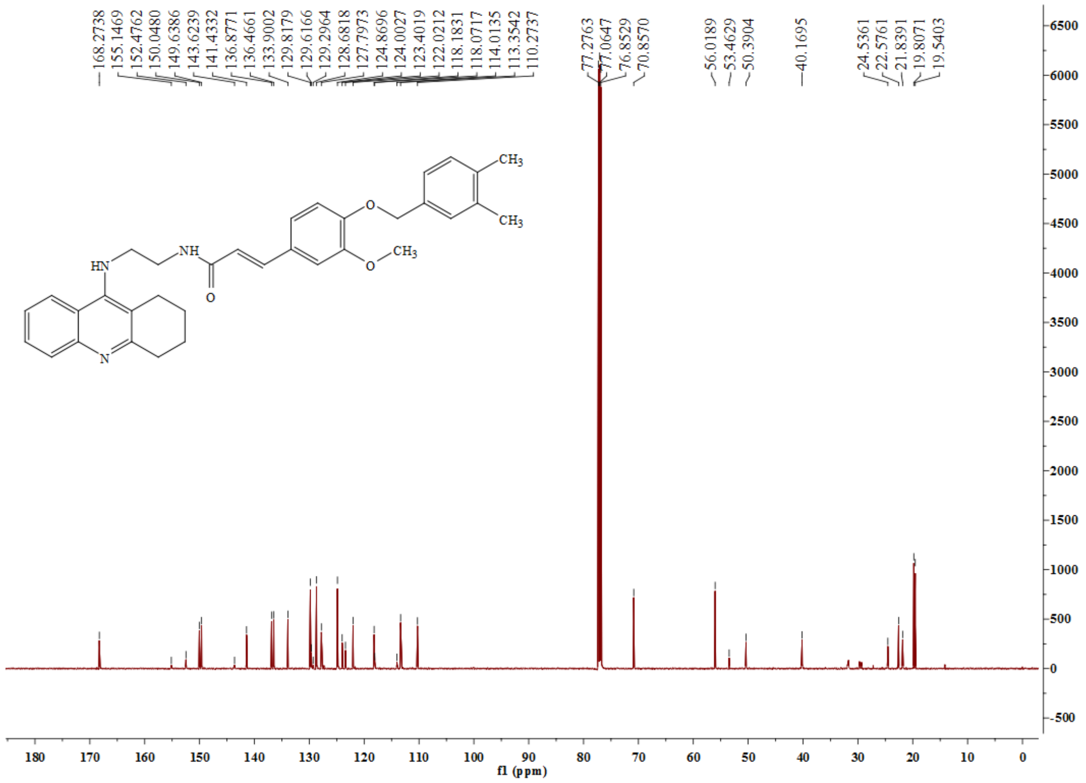

10h ^{1}H-NMR

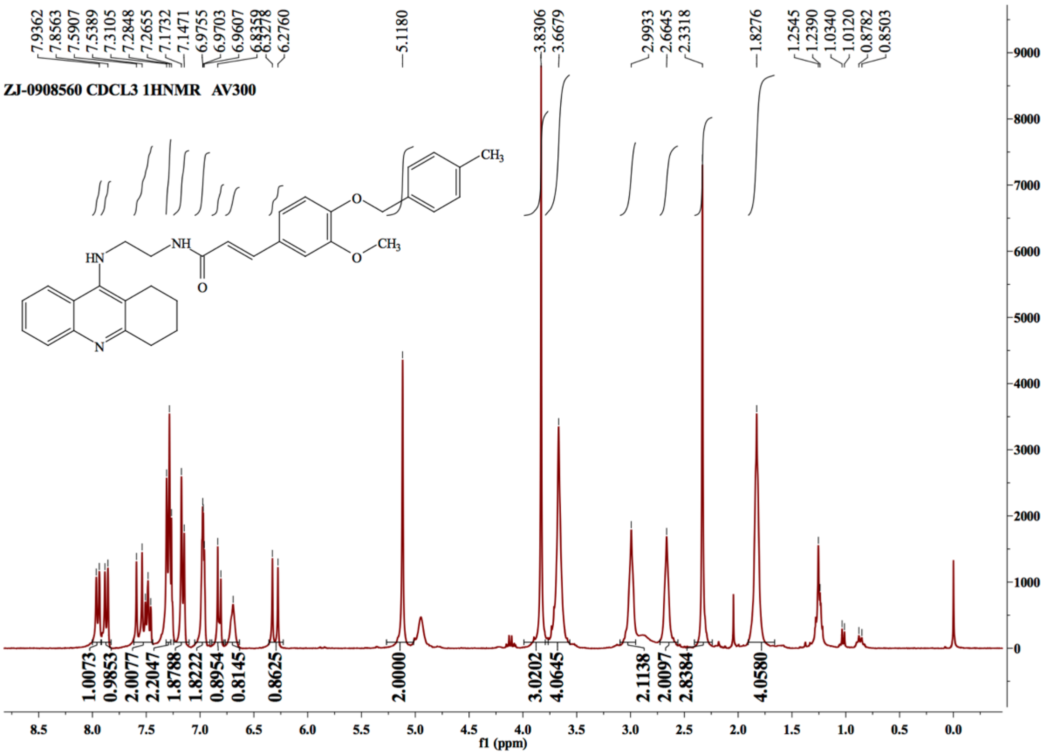

10h ^{13}C-NMR

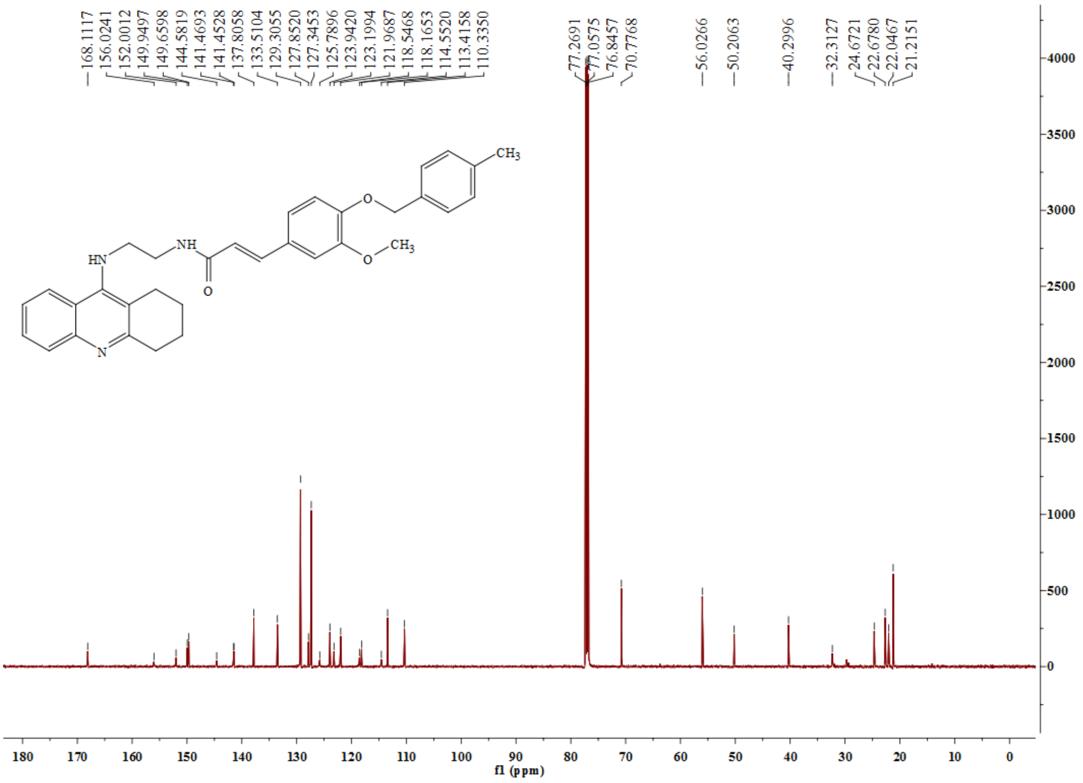

10i ^{1}H-NMR

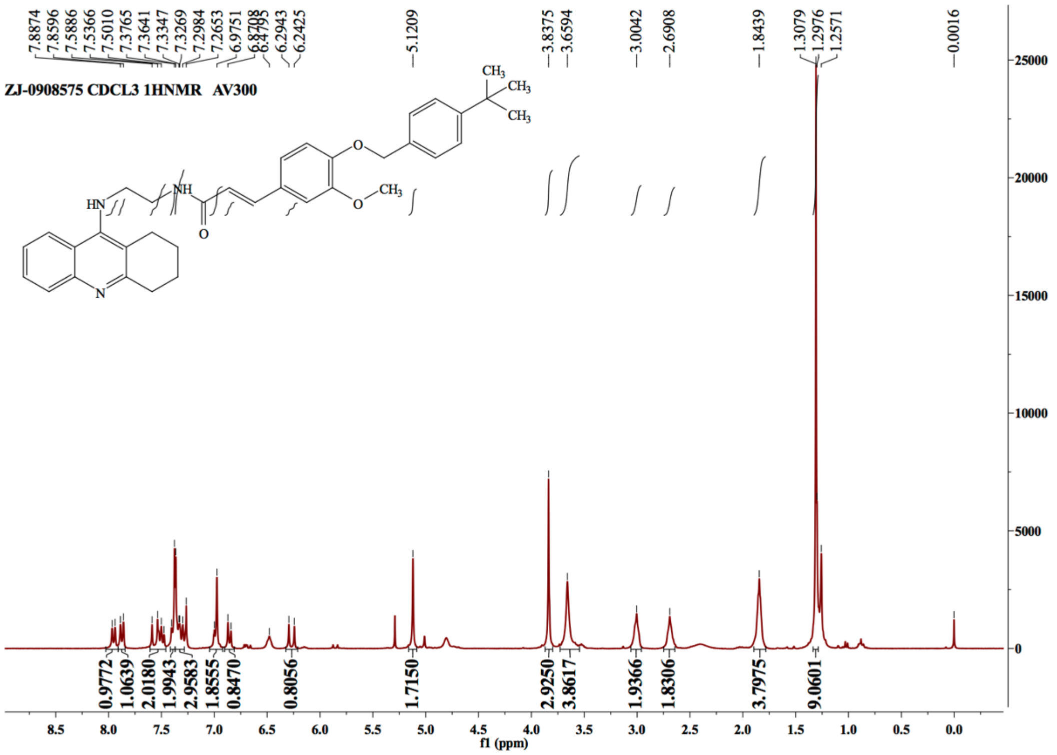

10i ^{13}C-NMR

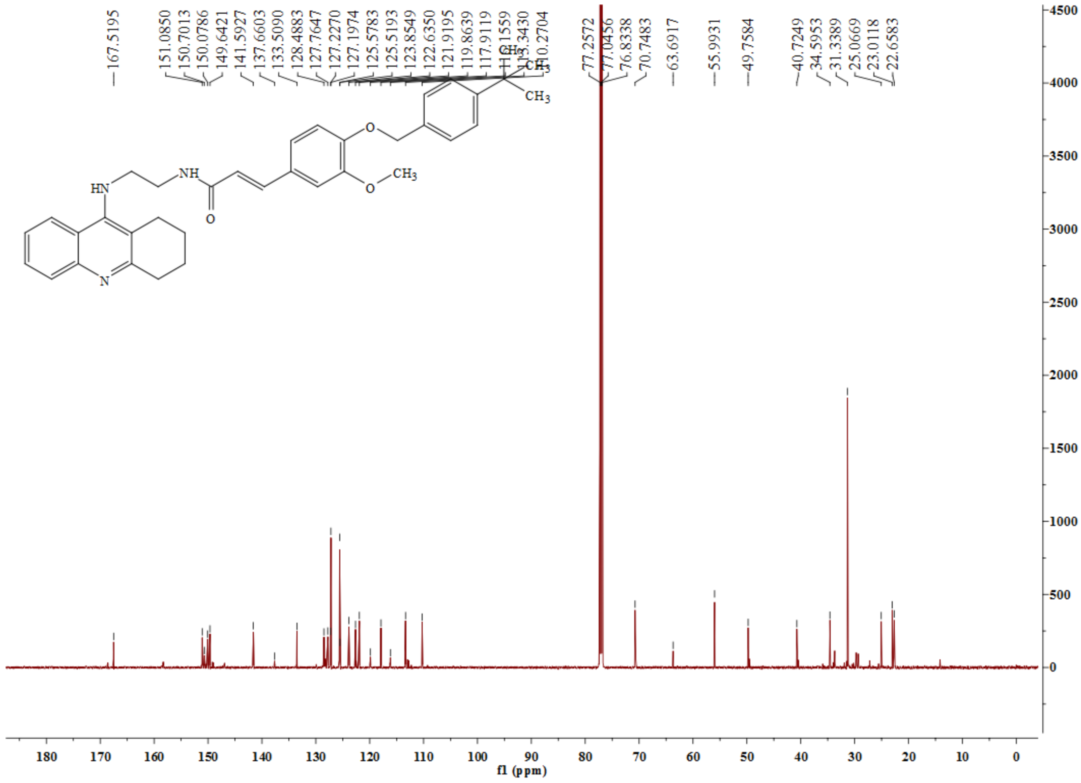

10j ^{1}H-NMR

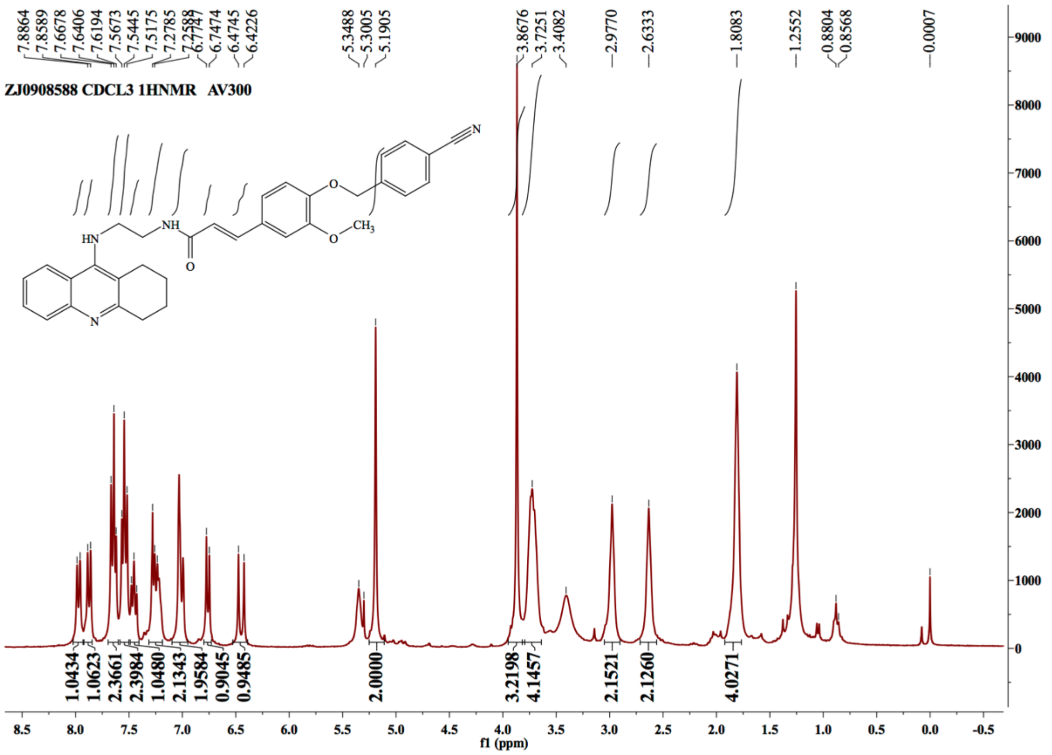

10j ^{13}C-NMR

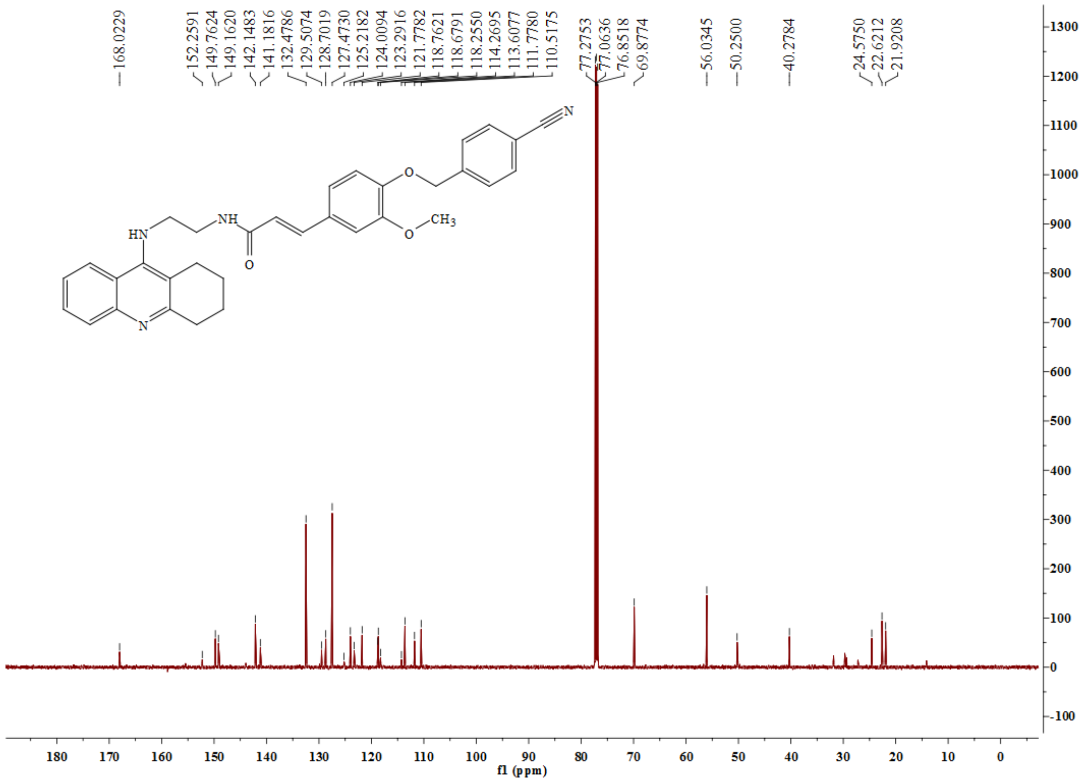

10k ^{1}H-NMR

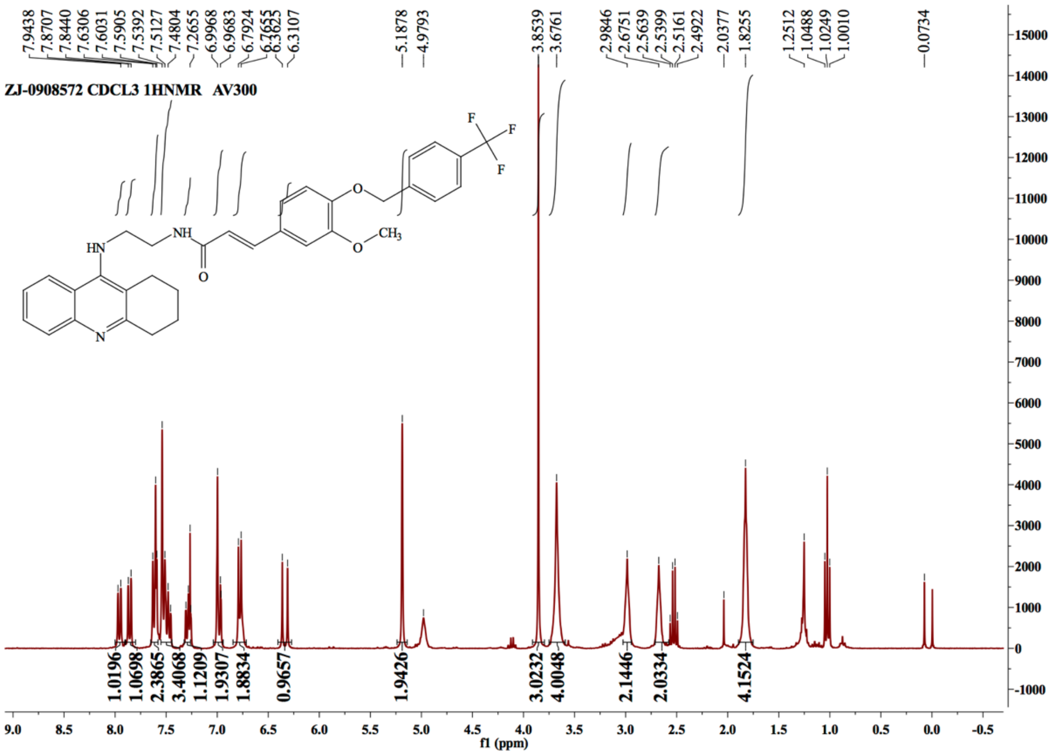

10k ^{13}C-NMR

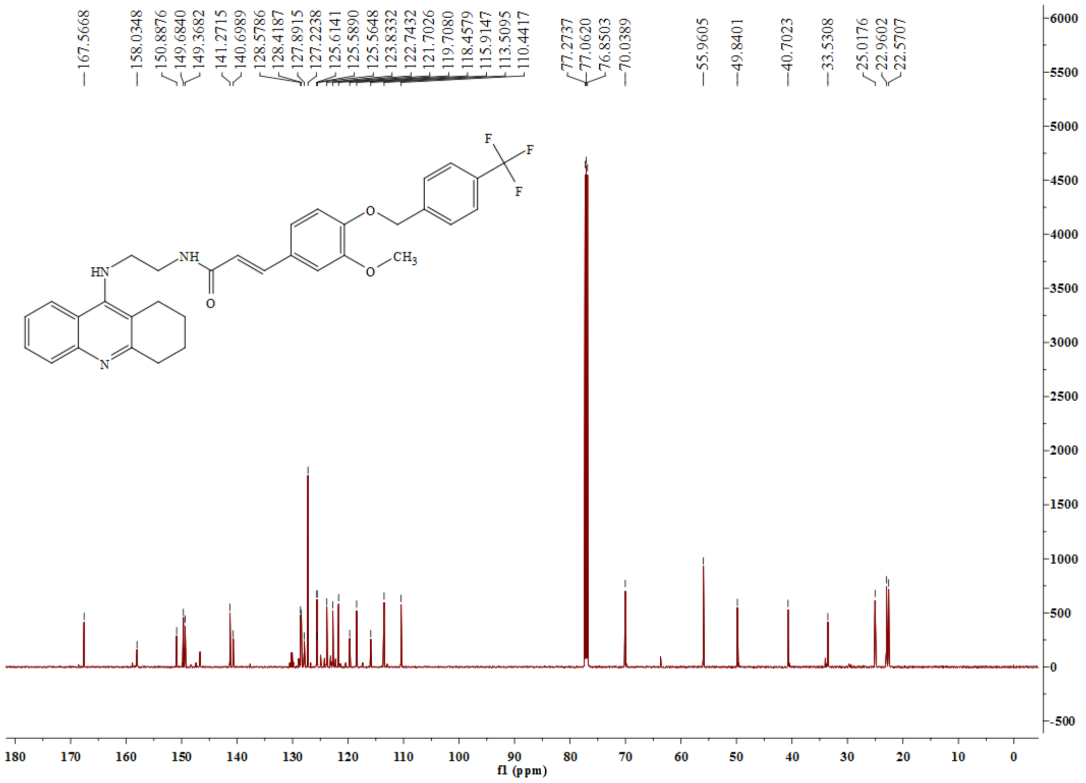

101 ^{1}H-NMR

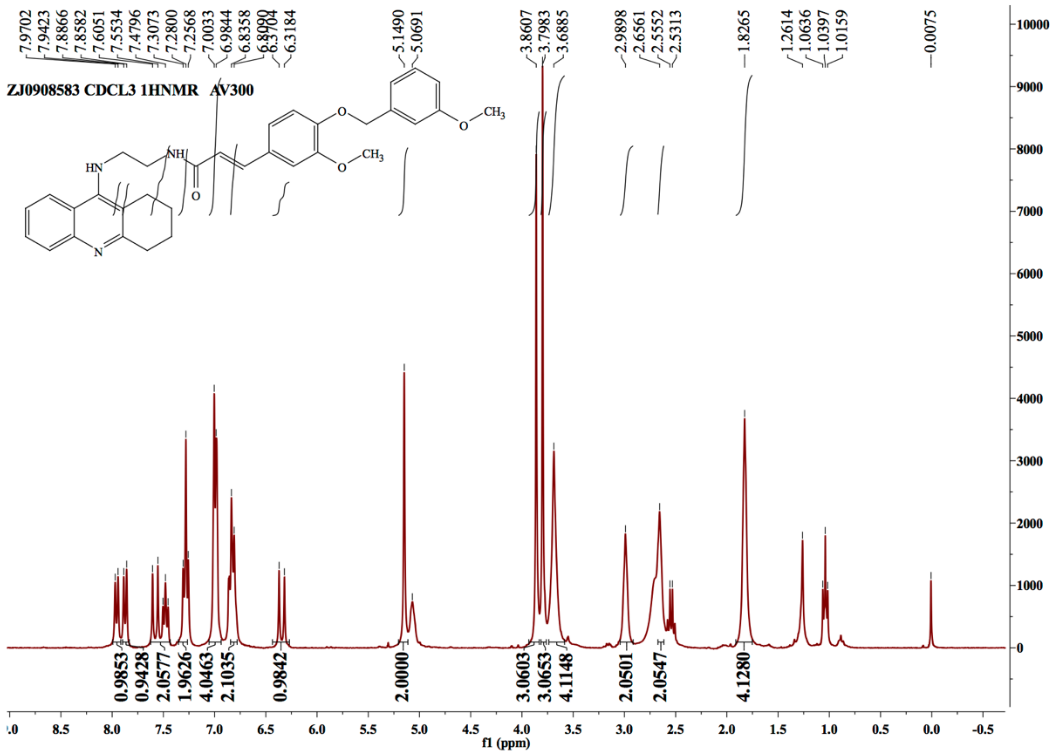

101 ^{13}C-NMR

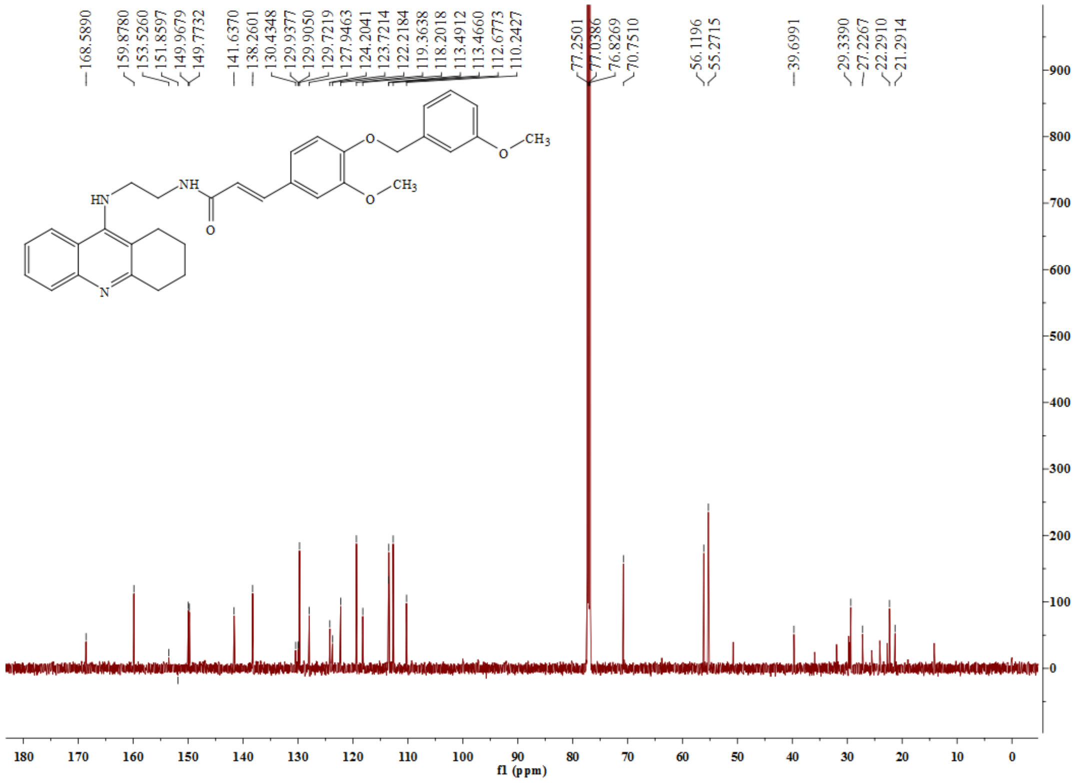

10m ^{1}H-NMR

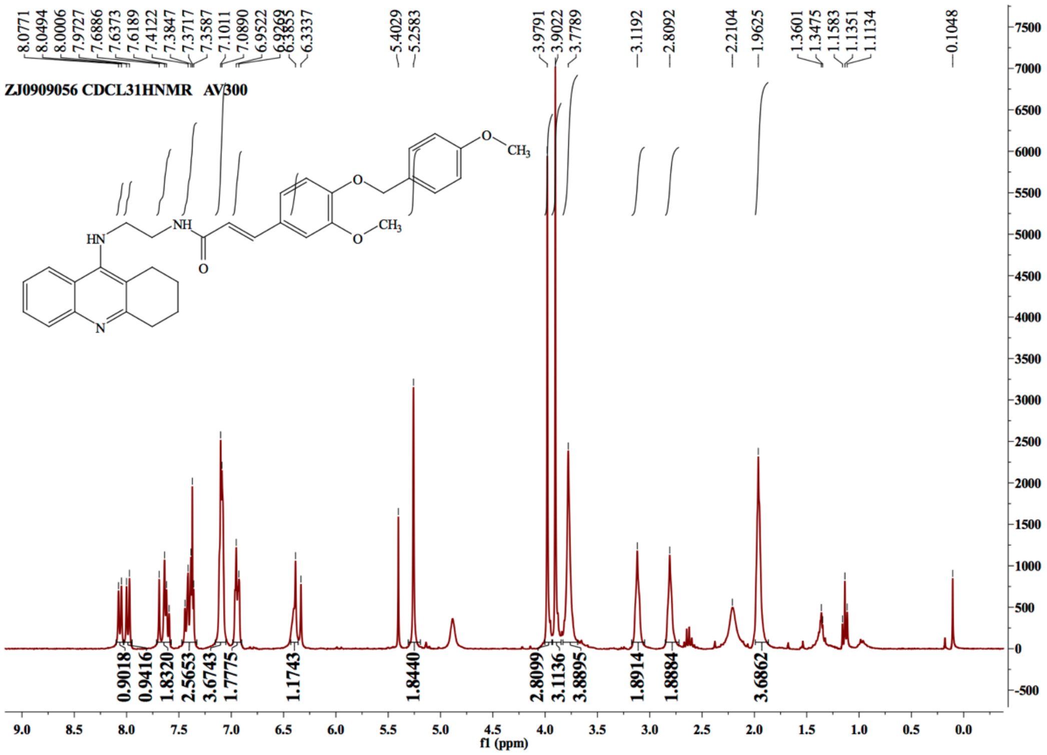

10m ^{13}C-NMR

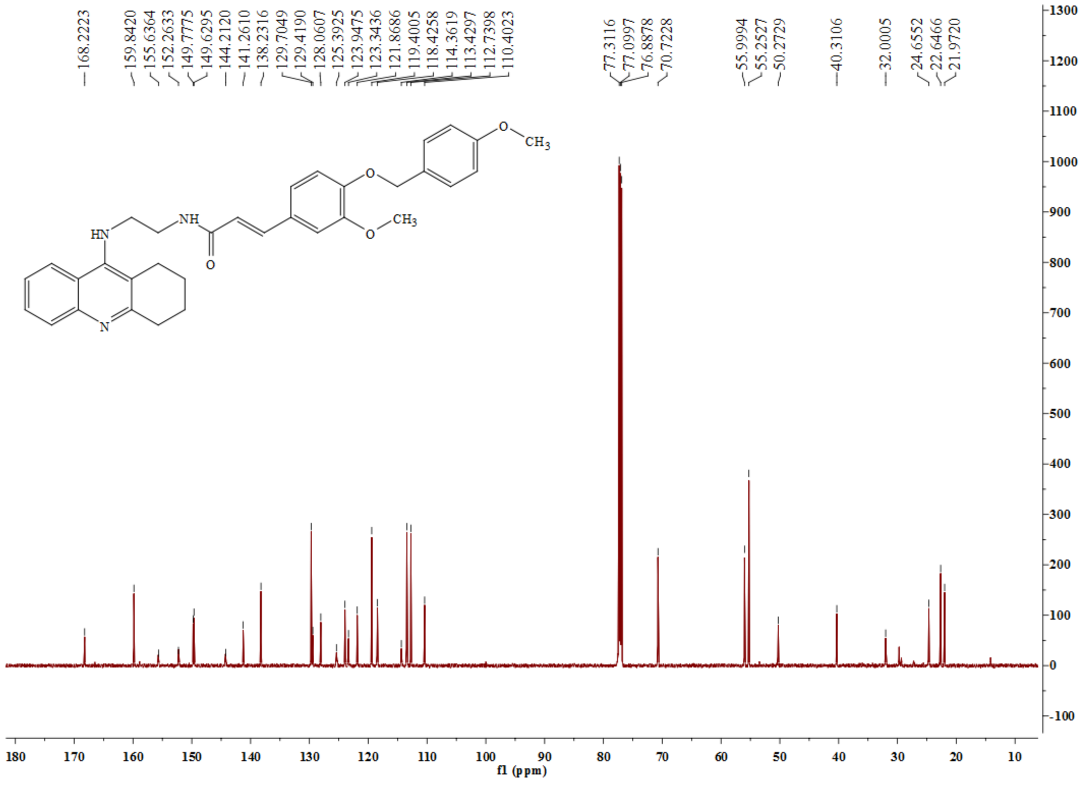

Table S1. The Cell viability of HepG2 cells after administration of different concentrations of **10d**, **10g** and tacrine.

Con. [b]	Cell viability (% of vehicle) [a]					
	0.05	0.1	0.5	2.5	5	50
**10d**	129.7 ± 7.5	125.0 ± 2.3	125.6 ± 2.1	130.6 ± 2.2	13.2 ± 2.3	2.5 ± 0.3
**10g**	128.4 ± 5.6	124.6 ± 1.7	121.7 ± 3.5	128.9 ± 4.1	1.5 ± 0.1	1.9 ± 0.3
Con. [b]	Cell viability (% of vehicle) [a]					
	25	125	250	500	1000	
Tacrine	106.2 ± 2.3	97.2 ± 2.9	58.9 ± 0.7	22.7 ± 1.6	11.0 ± 1.1	

[a] The results are presented as the percentage of viable cells observed after treatment with compounds vs vehicle-treated cells (100%) and are shown as the mean ± SEM, n = 3.

[b] concentration (μM).

Methods for Cell Viability Assay. The human hepatocellular HepG2 cells were cultured in RPMI 1640 medium. Cells were seeded into 96-well plates at a density of 3 × 10^4 cells per well and cultured in a humidified atmosphere of 95% air and 5% CO_2 at 37 °C for the following experiments. For cytotoxicity test, cells were treated with 0.05-50 μM per compounds and 25-1,000 μM of tacrine for 24h. Cell viability was measured by 3-(4,5- dimethylthiazol-2-yl)-2,5-diphenyltetrazolium bromide (MTT) analysis. In brief, 15 μL of MTT was added into each well and incubated at 37 °C for 4 h. The absorbance of each well was measured at 507 nm using a microplate reader. The cell viability was calculated and presented by the percentage of vehicle-treated group.
